# Occurrence of Food Chemical Contaminants in Entomophagy: Implications for Food Safety and Future Risk Assessment—A Scoping Review

**DOI:** 10.1111/1541-4337.70613

**Published:** 2026-08-19

**Authors:** Marta Esgalhado, Dayanne da Costa Maynard, Ariana Saraiva, Marjhory Gonçalves Moura, Didem Aybike Haspolat, José Costa, José Manuel Lorenzo, Zsuzsa Farkas, António Raposo, Maria do Céu Costa

**Affiliations:** ^1^ CBIOS (Research Center for Biosciences and Health Technologies), ECTS (School of Health Sciences and Technologies) Lusófona University Lisboa Portugal; ^2^ Department of Nutrition, Faculty of Health Sciences, Campus Universitário Darcy Ribeiro University of Brasília Brasília Brazil; ^3^ Research in Veterinary Medicine (I‐MVET), Faculty of Veterinary Medicine Lisbon University Centre, Lusófona University Lisboa Portugal; ^4^ Veterinary and Animal Research Centre (CECAV), Faculty of Veterinary Medicine Lisbon University Centre, Lusófona University Lisboa Portugal; ^5^ Department of Nutrition University Center of Brasília (CEUB) Brasília Brazil; ^6^ Nuh Naci Yazgan University Kayseri Türkiye; ^7^ Animal Feed Division/Nutrition and Food Services Directorate Directorate‐General for Food and Veterinary Affairs Lisboa Portugal; ^8^ Fundación Centro Tecnolóxico da Carne Ourense Spain; ^9^ Área de Tecnoloxía dos Alimentos, Facultade de Ciencias Universidade de Vigo Ourense Spain; ^10^ Department of Digital Food Science, Institute of Food Chain Science University of Veterinary Medicine Budapest Budapest Hungary; ^11^ Centro de Investigação do IPLuso (CIPLuso) Polytechnic Institute of Lusophony, ERISA—Escola Superior de Saúde Ribeiro Sanches Lisboa Portugal

**Keywords:** acrylamide, entomophagy, food safety, heavy metals, polycyclic aromatic hydrocarbons

## Abstract

Entomophagy is increasingly recognized as a sustainable alternative to conventional protein sources due to its low environmental impact. However, ensuring its safety requires careful evaluation of chemical contaminants of food safety concern. This scoping review aimed to map and synthesize the available evidence on the occurrence of food contaminants in edible insects, focusing on cadmium, lead, mercury, nickel, polycyclic aromatic hydrocarbons (PAHs), and acrylamide, and to discuss their implications for food safety and risk assessment. A systematic search was conducted in PubMed, Scopus, and gray literature up to April 2025, without date restrictions, following the PRISMA‐ScR guidelines. Only original research articles were included. Search terms addressed entomophagy and key contaminants, including cadmium, lead, mercury, nickel, acrylamide, and PAHs. Records were screened independently in Rayyan according to predefined eligibility criteria and relevance to the review question. Of the 1689 identified records, 114 duplicates were removed, 1575 were screened, and 23 underwent full‐text assessment. Five studies were excluded, and 8 additional articles were identified through manual searching, resulting in a total of 25 studies. The concentrations of the selected metal contaminants were generally low, although variability was observed across insect species and geographic origins. Exposure to PAHs and acrylamide was mainly influenced by rearing substrates and processing conditions. The available evidence suggests that insects produced under controlled conditions show contaminant levels that warrant continued monitoring and exposure‐based risk assessment. The absence of harmonized maximum contaminant levels for edible insects within European Union contaminant legislation highlights the need for additional data to support future regulatory and risk assessment frameworks.

## 1 Introduction

Insects have emerged as a sustainable and alternative source of high‐quality animal protein for both human consumption and livestock feed. Over the past decade, their production has gained considerable attention in Europe due to their efficient farming, environmental sustainability, and nutritional value. With the global population projected to exceed 9 billion by 2050 and the resulting increase in protein demand, insects are increasingly considered a promising substitute for conventional protein sources such as meat, crops, and fishmeal (Huis 2013; Jantzen da Silva Lucas et al. 2020).

Beyond their nutritional value, edible insects also contain bioactive compounds with potential health‐promoting properties (Rivero‐Pino et al. 2021; Sharma et al. 2022; Teixeira et al. 2023). These emerging applications further support the growing interest in edible insects as novel foods, reinforcing the need to ensure their chemical and microbiological quality throughout the food chain.

Despite these advantages, safety concerns remain. Insects can accumulate chemical contaminants from feed, water, or the environment (Marone 2016). Food contaminants, including cadmium (Cd), lead (Pb), mercury (Hg), and nickel (Ni), have been detected in both wild and farmed edible insects, with concentrations influenced by species, developmental stage, and rearing conditions (Green et al. 2001; Handley et al. 2007; Z. Zhang et al. 2012; Van Der Fels‐Klerx et al. 2018; Malejko et al. 2025). The European Food Safety Authority (EFSA) identifies contaminated feed as a primary source of these metals (EFSA Scientific Committee 2015), although controlled farming practices can reduce accumulation. Indeed, Poma et al. (2017) reported that farmed insects generally contain lower levels of metals than other commonly consumed animal products. Recent evidence indicates that chemical food safety hazards in edible insects are strongly influenced by the rearing substrate, insect species, developmental stage, and production conditions, highlighting the importance of appropriate farming practices and contaminant monitoring (Meyer et al. 2021).

Insects may also be associated with other chemical hazards beyond environmental contaminants. Mycotoxins produced by fungi such as *Fusarium*, *Aspergillu*s, and *Penicillium* may originate from contaminated feed or substrates (Lange and Nakamura 2021; Conway et al. 2024). In addition, thermal processing methods commonly applied to edible insect products, including roasting, baking, frying, and extrusion, may promote the formation of process‐induced contaminants such as acrylamide (Burešová et al. 2023; Bartkiene et al. 2023; European Commission 2017b) and polycyclic aromatic hydrocarbons (PAHs), compounds recognized for their potential genotoxic and carcinogenic properties (Burešová et al. 2023; Bartkiene et al. 2023; Cardoso et al. 2024; European Commission 2011b, 2023c). Chemicals used during farming, including biocides and veterinary drugs, may also persist in the final products, while certain insect species naturally produce toxic compounds (EFSA Scientific Committee 2015). To minimize microbiological and chemical risks, European Union (EU) regulations require a minimum 24‐h fasting period before killing and processing, allowing gut clearance and reducing potential contamination (European Commission 2017a). Microbiological hazards further complicate insect food safety. The high moisture and nutrient content of fresh insects promote microbial growth, emphasizing the need for appropriate decontamination and storage procedures (Rumpold and Schlüter 2013). Allergens and anti‐nutritional factors also require careful assessment to ensure a comprehensive safety profile, reinforcing the importance of strict control throughout production and processing.

Widely consumed species include caterpillars, bees, ants, crickets, and grasshoppers (Raheem, Carrascosa, et al. 2019; Raheem, Raposo, et al. 2019). Despite their nutritional value, consumer concerns about safety persist, particularly in low‐income regions with limited regulatory frameworks, even though insects are traditionally part of local diets and contribute significantly to nutrient intake (Imathiu 2020; Baiano 2020). These concerns highlight the need for further research on potential hazards such as toxins, allergens, and anti‐nutrients, as well as on microbiological risks and rapid spoilage of raw insect products, to guide the development of decontamination techniques and storage protocols.

Given the broad spectrum of potential hazards associated with edible insects, the present scoping review deliberately focused on a predefined group of chemical contaminants, namely, Cd, Pb, Hg, Ni, PAHs, and acrylamide. These contaminants were selected because they represent two major contamination pathways relevant to edible insects: environmental or substrate‐related accumulation (Cd, Pb, Hg, and Ni) and thermal processing‐induced contaminants (PAHs and acrylamide) (Imathiu 2020; EFSA Scientific Committee 2015; Van Der Fels‐Klerx et al. 2018). Other important hazards, including arsenic, mycotoxins, pesticide residues, veterinary drug residues, dioxins and polychlorinated biphenyls (PCBs), allergens, anti‐nutritional factors, and microbiological hazards, were considered outside the predefined scope of this review because they involve distinct sources of contamination, analytical methodologies, toxicological endpoints, and regulatory risk assessment frameworks, warranting separate dedicated evidence syntheses (EFSA Scientific Committee 2015; Imathiu 2020).

Ensuring food safety across the entire supply chain, from wild harvesting or farming to processing and storage, is critical for consumer confidence and the sustainable integration of insects into food systems. Recent reviews have further emphasized the importance of traceability, fraud prevention, and regulatory oversight throughout insect protein supply chains to strengthen consumer protection and facilitate the commercialization of insect‐based foods (Traynor et al. 2024; Carvalho et al. 2025). The lack of standardized value chain regulations in many countries, particularly in developing regions, poses challenges for both safety and trade (Imathiu 2020). Addressing these issues requires rigorous monitoring and testing protocols, together with clearly defined regulatory limits or product specifications for metal contaminants and other chemical hazards. Collaboration among scientists, policymakers, and industry stakeholders is essential to develop evidence‐based guidance, strengthen quality control, and support the sustainable production of insect‐based proteins while minimizing contaminant exposure (Niassy et al. 2022; Murefu et al. 2023).

This scoping review aims to map and synthesize the available evidence on the occurrence and potential food safety implications of six selected chemical contaminants in edible insects—Cd, Pb, Hg, Ni, PAHs, and acrylamide—while identifying current knowledge gaps and discussing considerations relevant to future risk assessment. Considering the growing industrial production and processing of insect‐based foods, these contaminants represent important toxicological concerns requiring further characterization. Accordingly, this work does not constitute a formal quantitative risk assessment as defined by EFSA, as it does not involve exposure modeling, margin of exposure (MOE) calculations, or full risk characterization. Instead, it provides an evidence‐mapping approach intended to identify data gaps, support hazard identification, summarize contaminant occurrence in relation to available regulatory and toxicological reference values, and inform future structured risk assessment frameworks incorporating dietary exposure assessment and risk characterization for the sustainable consumption of edible insects.

## 2 Methods

### 2.1 General Procedures

The objective of this study was to systematically identify, categorize, and synthesize the available literature on the occurrence of metal contaminants (Cd, Hg, Ni, and Pb) and process‐induced contaminants (acrylamide and PAHs) in edible insects. A scoping review was conducted following the Joanna Briggs Institute methodology for evidence synthesis, aiming to systematically map the existing evidence, characterize chemical contaminant occurrence, and identify research gaps relevant to future risk assessment approaches (Peters et al. 2020). The review protocol predefined the review objectives, eligibility criteria, search strategy, study selection process, data extraction, and evidence synthesis methods. The review was reported in accordance with the Preferred Reporting Items for Systematic Reviews and Meta‐Analyses for Scoping Reviews (PRISMA‐ScR) and registered on the Open Science Framework (OSF) (https://doi.org/10.17605/OSF.IO/928RY).

### 2.2 Information Sources and Search Strategies

Literature searches were performed in the electronic databases MEDLINE (via PubMed) and Scopus, as well as in the gray literature through Google Scholar. Additional studies were identified through manual screening of reference lists from selected articles. Searches were conducted on April 3 and 11, 2025. Search strategies were developed by two experts and refined through discussions with the entire research team. Descriptors were selected from the Medical Subject Headings (MeSH) and Health Sciences Descriptors (DeCS) and adapted for each database. Boolean operators were applied, with OR used to combine synonymous search terms within each concept and AND used to combine different concept groups. Search syntax was adapted to the indexing characteristics and search functionalities of each database. Full search strategies are provided in Table A1.

### 2.3 Research Question

This scoping review will be guided by the PCC framework (population, concept, and context), as follows:

- Population (P): Individuals consuming insect protein.
- Concept (C): Chemical contamination associated with insect protein, with emphasis on metal contaminants (Cd, Hg, Ni, and Pb) and process‐induced contaminants (acrylamide and PAHs), including their identification, characterization, and associated potential health risks.
- Context (C): The production and consumption of insect‐based foods, encompassing different production systems and contamination sources (natural vs. processing‐related), as well as comparisons with other animal protein sources.

Studies will be considered eligible if they are original research articles (observational or experimental) addressing chemical contaminants in insect protein.

Thus, the guiding research question for this scoping review was: “What is the available evidence on the occurrence of metal contaminants (Cd, Hg, Ni, and Pb) and process‐induced contaminants (acrylamide and PAHs) in edible insects, and how have these contaminants been characterized in relation to food safety and potential human health implications?”

### 2.4 Inclusion and Exclusion Criteria

This scoping review included original quantitative studies with observational or experimental designs, such as correlational, ecological, cohort, and cross‐sectional studies. Eligible studies were those that assessed the presence of chemical contaminants, namely, Cd, Hg, Ni, and Pb, as well as acrylamide and PAHs, in insect‐based products intended for human consumption. Studies were excluded if they (1) were not original quantitative research (e.g., literature reviews, scoping reviews, systematic reviews, editorials, letters to the editor, conference abstracts, opinion papers, books, dissertations, or theses); (2) used qualitative designs, such as interviews or case reports; (3) investigated insect species not intended for human consumption (e.g., insects used for animal feed, agricultural, or industrial purposes); (4) did not evaluate the target chemical contaminants (Cd, Hg, Ni, Pb, acrylamide, or PAHs) in insect‐based products intended for human consumption; or (5) assessed contamination under artificial experimental conditions, including studies using experimentally contaminated feed or intentionally elevated levels of contaminants or their precursors, as these conditions do not reflect real‐world production or processing scenarios.

Studies addressing arsenic, mycotoxins, pesticides, veterinary drug residues, dioxins, PCBs, allergens, anti‐nutritional factors, or microbiological hazards were also excluded because these hazards fell outside the scope of the review, which was restricted to six food chemical contaminants (Cd, Pb, Hg, Ni, PAHs, and acrylamide).

### 2.5 Study Selection

**FIGURE 1:**
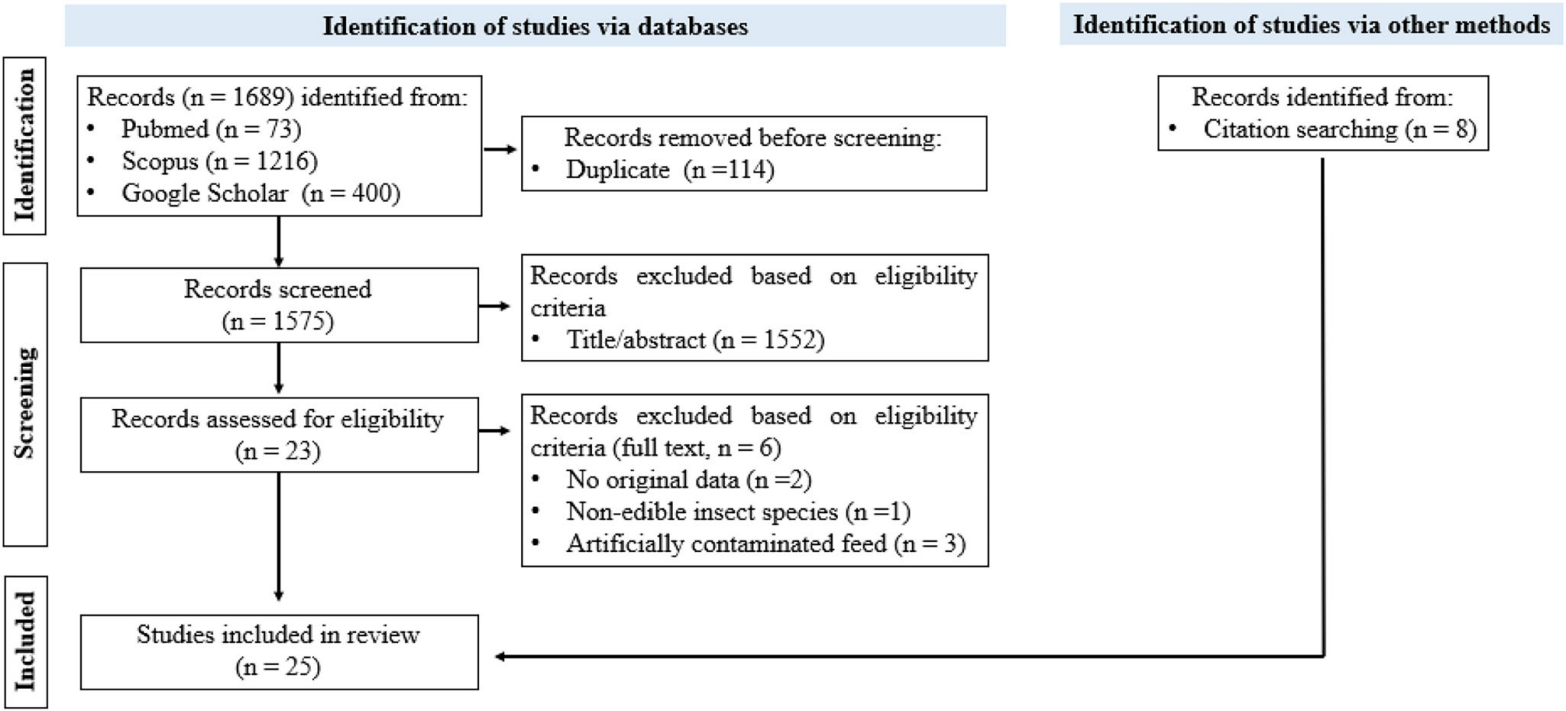
Flow diagram of the study selection process.

All studies were exported in RIS format and imported into the Rayyan application (Rayyan Qatar Computing Research Institute) for systematic review management. The study selection process (summarized in the PRISMA‐ScR flow diagram; Figure 1) followed a two‐phase approach to ensure consistency. In Phase 1, two reviewers independently screened titles and abstracts of all references identified in the databases against the predefined eligibility criteria. Articles that did not meet the eligibility criteria were discarded. In Phase 2, the same reviewers applied the eligibility criteria to the full texts of the selected articles to determine their final eligibility for inclusion. Reasons for exclusion at the full‐text stage were documented according to the predefined eligibility criteria and are summarized in Figure 1. In cases of disagreement, the matter was discussed in both phases until consensus was reached between the two reviewers. In situations where consensus was not reached, a third reviewer made the final decision.

### 2.6 Data Extraction

A structured data extraction table was developed in Excel 2013, including the following variables: year, author, country of study, title, objective, type of sample, insect species, origin, target contaminant, method of analysis for contaminant detection, main results, and legal reference (Table A2). To ensure consistency between reviewers, calibration exercises were performed before data extraction.

## 3 Results

Risk assessment metrics reported in the literature varied considerably across studies and included quantitative exposure and risk‐related approaches such as estimated daily intake (EDI), comparisons with tolerable daily intake (TDI) or tolerable weekly intake (TWI) values, target hazard quotient (THQ), MOE, and target cancer risk (TR) estimations. In most cases, exposure assessments were based on assumed consumption scenarios combined with measured contaminant concentrations. Some studies compared estimated intakes with established toxicological reference values to provide a preliminary indication of potential health risks. However, methodological heterogeneity was observed across studies, particularly regarding consumption assumptions, body weight parameters, the selection of toxicological reference values, and the reporting basis of contaminant concentrations. This variability limited the direct comparability of risk metrics between studies. Despite these methodological differences, the available evidence consistently applies risk assessment metrics as screening tools to contextualize contaminant occurrence rather than to establish definitive health risks. Within this scoping review, these approaches are described to characterize the range of risk assessment strategies reported in the literature, without performing independent quantitative risk characterization.

Whenever available, contaminant concentrations are reported using the basis specified in the original studies [dry weight (dw); wet weight (ww)]. When the original studies did not explicitly specify whether concentrations were expressed on a dry‐ or wet‐weight basis, the values are presented as provided in the original publications. An overview of the findings across the included studies, together with the number of studies and concentration ranges for each contaminant, is provided in Table 1. The results are presented below according to contaminant group.

**TABLE 1:** Summary of contaminant levels reported in studies included in the review.

Contaminant	Year	Number of studies	Insect species	Minimum value reported	Maximum value reported	Comparative regulatory/toxicological reference values
Pb	2009–2025	21	*Tenebrio molitor*, *Acheta domesticus*, *Locusta migratoria*, *Alphitobius diaperinus*, *Bombyx mori*, *Schistocerca gregaria*, *Zonocerus variegatus*, *Hermetia illucens*	0.003 mg/kg	0.501 mg/kg	0.1 mg/kg (Commission Regulation (EU) 2023/915, comparative regulatory benchmark); 2 mg/kg (FAO/WHO guideline)
Ni	2017–2025	7	*T. molitor*, *A. domesticus*, *L. migratoria*, *A. diaperinus*, *B. mori*, *H. illucens*, *Galleria mellonella*, *S. gregaria*	0.19 mg/kg	2.23 mg/kg	13 µg/kg bw/day (EFSA TDI)
Hg	2009–2023	13	*T. molitor*, *A. domesticus*, *L. migratoria*, *B. mori*, *H. illucens*, *A. diaperinus*, *G. mellonella*, *Grasshopper* spp.	0.00094 mg/kg	2.04 mg/kg	0.5 mg/kg (Commission Regulation (EU) 2023/915, comparative regulatory benchmark)
Cd	2009–2025	21	*L. migratoria*, *T. molitor*, *A. domesticus*, *A. diaperinus*, *H. illucens*, *B. mori*, *Macrotermes bellicosus*	0.01 mg/kg	0.1–2 mg/kg	0.05–0.2 mg/kg (Commission Regulation (EU) 2023/915, comparative regulatory benchmark values for selected food categories)
PAHs	2015–2020	2	*Musca domestica*, *Calliphora vomitoria*, *Chrysomya* spp., *H. illucens*	0.001 mg/kg	2 mg/kg	0.001 mg/kg (BaP); 0.005 mg/kg (PAH4) (Commission Regulation (EU) 2023/915, comparative regulatory benchmark values)
Acrylamide	2023–2024	3	*A. domesticus*, *T. molitor*	14.03 µg/kg	68.78 µg/kg	50 µg/kg (Commission Regulation (EU) 2017/2158, benchmark level)

*Note*: Regulatory benchmark values are presented as comparative reference values and are not necessarily specific maximum limits established for edible insects.

Abbreviations: BaP, benzo[*a*]pyrene; EFSA, European Food Safety Authority; EU, European Union; FAO, Food and Agriculture Organization; PAH4, sum of four priority polycyclic aromatic hydrocarbons; PAHs, polycyclic aromatic hydrocarbons; TDI, tolerable daily intake; WHO, World Health Organization.

### 3.1 Metals

#### 3.1.1 Cadmium

The included studies comprised both experimentally reared insects under controlled production conditions and commercially available insect products, which may differ in their potential sources of Cd contamination. These differences should be considered when interpreting the reported concentrations.

Cd was the most extensively investigated metal contaminant, with studies conducted across Europe, Africa, Asia, and North America. Despite substantial heterogeneity in insect species, production systems, rearing substrates, geographical origin, reporting units, and exposure assessment approaches, several consistent patterns emerged regarding Cd occurrence, the influence of production systems, environmental exposure, and processing practices on Cd accumulation, as well as the associated dietary risk (Charlton et al. 2015; Poma et al. 2017; Truzzi et al. 2019; Köhler et al. 2019; Van Der Fels‐Klerx et al. 2020; Kolakowski et al. 2021; Kosečková et al. 2022; Muhammad et al. 2022; Murefu et al. 2023; Mwelwa et al. 2023; Holowaty et al. 2024; Gori et al. 2025).

In Europe, Gori et al. (2025) analyzed 52 insect‐based products commercialized on European e‐commerce platforms. These products were 16 powders/whole insects, 15 salty snacks, 15 sweet snacks, and 6 pastas. Only the 10 products composed entirely of insect material (powder or whole insects) were compared with the tolerable limits established by the relevant Commission Implementing Regulations (CIRs), according to insect species and food category (frozen, dried, or powdered): 2023/58 (European Commission 2023a) (≤ 0.05 mg/kg) for *Alphitobius diaperinus*, (EU) 2022/188 (European Commission 2022b) (≤ 0.06 mg/kg) and (EU) 2023/5 (European Commission 2023b) (≤ 0.025 mg/kg) for *Acheta domesticus*, (EU) 2021/1975 (European Commission 2021a) (≤ 0.05 mg/kg) for *Locusta migratoria*, and (EU) 2021/882 (European Commission 2021b) (≤ 0.1 mg/kg) and (EU) 2022/169 (European Commission 2022a) (≤ 0.1/≤ 0.05 mg/kg) for *Tenebrio molitor*. The results indicated that one *T. molitor* sample exceeded the Cd limit. Four samples exceeded the maximum levels established for animal‐derived raw materials (0.05 mg/kg) as defined by Commission Regulation (EU) No 2023/915 (European Commission 2023c). Nevertheless, all measured Cd concentrations remained below the regulatory benchmark values established for crustaceans (0.50 mg/kg), cephalopods (1.0 mg/kg), and bivalve mollusks (1.0 mg/kg) under Commission Regulation (EU) No. 2023/915 (European Commission 2023c), which served as comparative reference values in the absence of harmonized maximum levels for edible insects. Despite these exceedances, the estimated weekly intake of Cd from consumption of these products remained low, between < 0.01% and 7.86% of the TWI of 2.5 µg/kg body weight/week established by the EFSA Panel on Contaminants in the Food Chain (CONTAM) (2011), indicating limited dietary exposure under the assumed consumption scenario. Cumulative dietary exposure should still be considered, especially in frequent consumers and vulnerable populations such as children. Truzzi et al. (2019) investigated *T. molitor* larvae, which were reared for 4 months on organic wheat flour or organic wheatmeal, used either alone or combined with increasing proportions of organic olive pomace (25%, 50%, and 75%). Cd concentrations ranged from 0.008 to 0.016 mg/kg ww, with organic wheatmeal presenting the highest levels and olive pomace presenting the lowest. No statistically significant correlation was observed between Cd content in the feed and in the larvae, and the bioaccumulation factor (BAF) approximated 1, indicating minimal bioaccumulation. Cd concentrations remained below the maximum levels established under current EU legislation for contaminants in foodstuffs (European Commission 2023c). Similarly, Poma et al. (2017) analyzed larvae of *Galleria mellonella*, adults of *L. migratoria*, larvae of *T. molitor*, and larvae of *A. diaperinus*, as well as insect‐based products such as locust‐ and buffalo worm‐based “bugballs,” cricket croquettes, and buffalo worm‐based “bugburger” commercialized in Belgium. Cd was detected in all samples at levels below 0.06 mg/kg ww, remaining well below the maximum levels established under EU legislation for contaminants in foodstuffs (European Commission 2023c). Holowaty et al. (2024) analyzed commercially available dried insects from both the French retail market and online vendors, including crickets (*A. domesticus*), mealworms (*T. molitor*), ants (*Atta laevigata*), water bugs (Nepidae), locusts (*L. migratoria*), scorpions (*Heterometrus longimanus*), and tarantulas (*Haplopeima albostriatum*). Using the Joint FAO/WHO Expert Committee on Food Additives (JECFA 2011) provisional tolerable monthly intake (PTMI) for Cd of 25 µg/kg body weight/month (equivalent to approximately 0.82 µg/kg body weight/day), the maximum estimated daily consumption corresponding to this toxicological reference value was calculated for a 60 kg adult and an 18 kg child. The estimated intake was interpreted considering international toxicological guidance values established by FAO/WHO‐JECFA and EFSA for Cd exposure (JECFA 2011, EFSA Panel on Contaminants in the Food Chain (CONTAM) 2011). Based on these estimates, most insects, particularly crickets, mealworms, and locusts, could be consumed in relatively large quantities before reaching the PTMI‐derived reference intake (approximately 1000–100,000 individuals), whereas scorpions had a markedly lower estimated consumption threshold (12–32 for adults; 4–10 for children). Locusts also had a lower estimated consumption threshold than most other insects (≈2225 for adults), demonstrating that Cd content can vary considerably among species and may influence estimated dietary exposure. Van Der Fels‐Klerx et al. (2020) evaluated Cd accumulation in *Hermetia illucens* larvae reared on substrates containing 3%–6% shredded packaging material. Four substrate types were tested: meat‐based plastic packaging, vegetarian plastic packaging, meat‐based carton packaging, and vegetarian carton packaging. Larvae accumulated Cd up to 9.5 ± 3.6‐fold when reared on substrates containing Cd at 0.5 times the EU maximum level permitted for animal feed (0.25 mg/kg) (European Commission 2002), demonstrating that contaminated substrates can significantly increase Cd concentrations in *H. illucens* larvae.

Muhammad et al. (2022) analyzed grasshoppers (*Zonocerus variegatus*), fingerlings (*Oreochromis niloticus*), termites (*Macrotermes bellicosus*), and locusts (*Schisocerca gregaria*) collected in Dutsin‐Ma, Nigeria. *Z. variegatus* exhibited the highest Cd content (7.11 ± 0.89 mg/kg), followed by *O. niloticus* (5.78 ± 0.75 mg/kg), *Schistocerca gregaria* (3.51 ± 1.62 mg/kg), and *M. bellicosus* (1.74 ± 0.00 mg/kg). Since THQ is generally associated with noncarcinogenic risk assessment, not carcinogenic risk, THQ > 1 means potential noncarcinogenic health concerns and possible adverse health effects from chronic exposure (USEPA 2011). The THQ exceeded the safety threshold of 1 in several species, particularly for children, suggesting potential health concerns associated with chronic Cd exposure. In *Z. variegatus*, the THQ was 1.16 in adults and 4.05 in children, while *O. niloticus* showed THQ values of 0.79 and 2.77, respectively. In addition, *M. bellicosus* had values of 0.28 in adults and 0.99 in children, while *S. gregaria* had values of 0.57 and 2.00, respectively. Furthermore, the TR values for Cd were classified as moderate, according to the New York State Department of Health (New York State Department of Health 2007). This suggests a nonnegligible probability of cancer development with prolonged exposure. Badanaro et al. (2022) investigated 12 edible insect species from Togo belonging to the orders Coleoptera (*Cybister tripunctatus*, *Gnathocera trivittata*, *Gnathocera impressa*, *Gnathocera varians*, *Rhabdotis sobrina*, *Pachnoda cordata*, *Pachnoda marginata*, and *Sternocera interrupta*), Isoptera (*M. bellicosus*), and Orthoptera (*Acanthacris ruficornis*, *Kraussaria angulífera*, and *Brachytrupes membranaceus*). Cd concentrations ranged from 0.016 to 0.504 mg/kg, with the highest values observed in *C. tripunctatus*, slightly exceeding the EU maximum limit of 0.5 mg/kg (European Commission 2006). These findings indicate that most species remained below the selected regulatory benchmark values, whereas occasional exceedances, such as in *C. tripunctatus*, highlight the need for monitoring, particularly when insects are consumed frequently or in large quantities. Environmental exposure plays a pivotal role in Cd accumulation, as demonstrated by Mwelwa et al. (2023), who investigated Cd biotransfer along the soil–plant–edible insect–human food chain in a mining‐impacted region of Zambia. The study quantitatively determined Cd concentrations in soils, host plants, and commonly consumed insects, including caterpillars, termites, and grasshoppers, across a 60 km gradient from the mining site (sampling at 1.5, 16.5, 31.5, 46.5, and 61.5 km). Cd levels in soils were highest at sites closest to the mining activities (0.006 µg/mg at 1.5 km) and gradually decreased with distance, reaching 0.002 µg/mg at 61.5 km. This spatial trend was reflected in insect samples, indicating that environmental contamination strongly influences Cd accumulation in edible insects and, consequently, potential human exposure. Post‐harvest processing can substantially reduce Cd exposure in edible insects, as reported by Murefu et al. (2023). Mopane caterpillars (*Gonimbrasia belina*) initially contained 4.59–5.88 mg/kg Cd, which decreased dramatically to 0.04 mg/kg following charcoal roasting and sun‐drying. These findings highlight the critical role of appropriate processing methods in mitigating heavy metal risks associated with insect consumption. Kolakowski et al. (2021) analyzed crickets (*n* = 15) and silkworm pupae (*n* = 4) sourced from Canadian markets, including whole dried insects, powders, and insect‐based products such as protein bars. Cd was undetectable in all silkworm pupae‐based products, whereas cricket‐based samples consistently contained measurable Cd concentrations ranging from 0.031 to 0.23 mg/kg, with a mean value of 0.083 mg/kg. These findings contrast with earlier studies reporting Cd concentrations below 0.03 mg/kg in edible insects, suggesting that both insect species and product form may influence Cd exposure. Although specific regulatory limits for edible insects remain limited, the detected concentrations were generally within the range established under EU legislation for contaminants in foodstuffs (European Commission 2023c). Other studies integrating multiple continents include Charlton et al. (2015), who analyzed fly larvae (*Musca domestica*, *Calliphora vomitoria*, *Chrysomya* spp., and *H. illucens*) from the United Kingdom, Ghana, Mali, and China. Cd was detected in all dried insect samples, with the highest concentrations observed in *M. domestica* (from 625 to 723 µg/kg), exceeding the maximum level of 500 µg/kg established for animal feed by Directive 2002/32/EC (European Commission 2002). Cd concentrations in the other species remained below this limit. The authors attributed the elevated Cd levels in *M. domestica* to environmental pollution, industrial activity, and agricultural practices in the regions of origin, underscoring the need for maximum Cd concentrations in insect feedstock to mitigate contamination along the food chain.

Köhler et al. (2019) analyzed four insect species: Bombay locust *Patanga succincta*, scarab beetle *Holotrichia* sp., house cricket *A. domesticus*, and mulberry silkworm *Bombyx mori*. All four species were purchased from a street hawker (SH) in a well‐known tourist area. In addition, cricket and silkworm samples (15 g net weight each, in original‐flavor foil packs) were obtained from a supermarket (SM) in downtown Bangkok. Cd levels in all the insect samples were below 0.005 mg/100 g. The heavy metal content of all the insect samples was below the applicable maximum regulatory limits for feed materials and complete feed. No significant difference was observed in Cd content between insects purchased from street vendors and those obtained from SMs. Heavy metal accumulation was low, and Cd levels remained below the applicable regulatory limits, supporting compliance with the relevant contaminant standards for the analyzed samples. Sikora et al. (2023) reported that in 2021, 14 insect‐based products were purchased from online stores registered in the EU. These included the buffalo worm *A. diaperinus*, yellow mealworm *T. molitor*, house cricket *A. domesticus*, migratory locust *L. migratoria*, and domesticated silkworm *B. mori*. Twelve products were intended for human consumption, while two were designated animal feed. Most products were based on insect species registered as novel foods within the EU. However, the silkworm *B. mori* product, based on insects not regulated under EU law, was still available for purchase from a registered retailer. Three replicates were prepared from each sample, each weighing 1.000 g. The median Cd level in the analyzed insects was 0.0026 mg/100 g. In addition, all insect species remained consistently below the maximum permitted level for Cd established by EFSA. This indicates that commercially available edible insect‐based products in the EU generally comply with the applicable regulatory limits with respect to contamination by toxic metals, metalloids, and rare earth elements. On the other hand, Kosečková et al. (2022) analyzed a total of 14 samples, including commercially available cricket powder (*n* = 3) and farmed insect species: house cricket *A. domesticus* adults (*n* = 3), yellow mealworm *T. molitor* larvae (*n* = 3), desert locust *S. gregaria* adults (*n* = 2), and superworm *Zophobas morio* larvae (*n* = 3). Samples were purchased from retail SMs and pet shops in the South Moravian region. Cd levels in edible insect samples (mg/100 g dw) were reported as follows: cricket powder (made from *A. domesticus*) ranged from 0.002 to 0.01 mg/100 g, house cricket (*A. domesticus*) from 0.004 to 0.006 mg/100 g, mealworm (*T. molitor*) from 0.008 to 0.014 mg/100 g, desert locust (*S. gregaria*) from 0.003 to 0.008 mg/100 g, and superworm (*Z. morio*) from 0.002 to 0.003 mg/100 g. Although maximum regulatory levels are typically established on a fresh weight (fw) basis, the dw values reported in this study remained below the selected regulatory benchmark values, indicating that Cd concentrations were lower than these benchmarks.

In addition, another study Marzoli et al. (2023) analyzed *B. mori* and its derived products, including chrysalises, chrysalis oil, and defatted chrysalis meal. Cd was detected in all samples, ranging from 0.021 to 0.45 mg/kg, with the highest concentration observed in defatted chrysalis meal, highlighting that processing can modulate heavy metal content in silkworm‐derived products. Cd was detected in all samples, starting from < 0.005 mg/kg, with very low levels observed in chrysalides, chrysalis oil, and chrysalis meal. In *B. mori*, potential Cd contamination via soil, feed, air, and handling‐related (manipulation) sources, as well as microbial and chemical pathways, remained below the applicable regulatory limits.

In Asia, specifically in China, Z.‐S. Zhang et al. (2009) investigated Cd dynamics along the soil–plant–insect food chain, demonstrating that plants act as the primary accumulation point. Soil Cd concentrations were substantially elevated, up to 149‐fold higher than background values, resulting in pronounced accumulation in plants, with a concentration factor (CF) of 6.82 and a mean concentration of 203.4 mg/kg, although with considerable variability among species. This indicates that Cd is readily transferred from soil to plants, representing one of the most efficiently accumulated metals in this subsystem. Subsequently, Cd transfer to insects was more limited, with CF values of 2.01 in herbivorous insects and 0.48 in carnivorous insects, indicating a progressive decrease along trophic levels. No significant differences in Cd concentrations were observed among insect species (*F* = 1.616, *p* = 0.212), suggesting a relatively uniform exposure at this level. Consistent with this, Kim et al. (2020) investigated *L. migratoria* collected from farms in Jangseong, South Korea, comparing specimens reared on corn versus wheat. Detectable Cd levels (0.03 mg/kg) were found in corn‐fed locusts, whereas no Cd was detected in those fed wheat. These results underscore the significant role of feeding substrate in modulating Cd accumulation, likely reflecting underlying differences in soil and plant contamination associated with each feed source. Nevertheless, all measured concentrations remained within the established regulatory limits for edible insects in South Korea (0.05–0.3 mg/kg), as reported in the study. Similarly, Hyun et al. (2012) reported low Cd concentrations (0.002–0.005 mg/100 g) in *Oxya chinensis formosana* (grasshopper) under controlled rearing conditions in South Korea, further supporting the role of production systems in minimizing contamination. Soren et al. (2021) analyzed *Tarbinskiellus portentosus* and *Schizodactylus monstrosus*, consumed by the Bodo tribe in Assam, India. The results indicated Cd levels of 0.015 ppm in *T. portentosus* and 0.004 ppm in *S. monstrosus*. Although no regulatory limits were applied, the authors suggested that these values may reflect environmental contamination and advised caution. Kitahara et al. (2022) assessed 14 edible insect species available in Japan, including beetles, crickets, silkworm pupae, and scorpions. Cd was detected in 79% of the samples, with median and maximum concentrations of 0.01 and 0.82 ppm, respectively. The highest Cd concentration was identified in the Asian (Wang et al. 2025) forest scorpion (0.82 ppm), exceeding the established Cd limit for rice (0.4 ppm), although according to the standards set by the Food and Agriculture Organization (FAO), Cd concentrations exceeding 0.2 mg/kg in rice could pose a risk to humans (JEFCA 2011). Nevertheless, the authors emphasized that the relatively low consumption frequency of this species mitigates the immediate risk to human health.

The available evidence consistently indicates that Cd concentrations in edible insects are generally low under controlled farming conditions and usually remain within current regulatory comparator values. Variability between studies was primarily associated with environmental contamination, rearing substrate, insect species, and, to a lesser extent, processing practices. Quantitative exposure assessments generally indicated limited dietary exposure under typical consumption scenarios, despite isolated exceedances, supporting the need for continued monitoring as edible insect production and commercialization continue to expand (Poma et al. 2017; Truzzi et al. 2019; Van Der Fels‐Klerx et al. 2020; Kolakowski et al. 2021; Kosečková et al. 2022; Murefu et al. 2023; Mwelwa et al. 2023; Holowaty et al. 2024; Gori et al. 2025).

#### 3.1.2 Nickel

Ni concentrations were investigated in both commercially available insect products and farmed edible insects, with additional evidence from environmentally contaminated areas and post‐harvest processing studies (Gori et al. 2025; Truzzi et al. 2019; Mwelwa et al. 2023; Murefu et al. 2023). Although Ni concentrations vary according to insect species, product formulation, and environmental conditions, the available evidence generally indicates low accumulation under controlled farming conditions and limited dietary exposure (Poma et al. 2017; Kosečková et al. 2022; Sikora et al. 2023).

In the European market, Gori et al. (2025) analyzed insect‐based products sold through e‐commerce, including powders and whole insects. Products with higher insect content (51%–90%) exhibited the highest Ni concentrations, with a mean value of 2.23 ± 0.3 mg/kg, whereas products with lower insect content ranged from 0.42 to 0.69 mg/kg. These differences could not be solely attributed to the insect ingredient, suggesting that additional components or processing factors may influence Ni levels. Although no regulatory limits existed for Ni in foods at the time of most of the reviewed studies, the observed concentrations exceeded those typically reported in animal‐derived products and were comparable to Ni‐rich vegetables and legumes. EDI values reached up to 7.38% of the TDI established by EFSA (13 µg/kg body weight/day) (Schrenk et al. 2020), with the highest exposure observed in young children consuming products containing *L. migratoria*. Notably, 12.3% of the MOE values fell below 30, indicating potential concern for sensitive populations, particularly for samples derived from *T. molitor*, *A. domesticus*, *L. migratoria*, and *A. diaperinus*. Since then, Commission Regulation (EU) 2024/1987 has established maximum levels for Ni in certain foodstuffs by amending Regulation (EU) 2023/915 (European Commission 2023c); however, no specific maximum levels have yet been established for edible insects.

In controlled farming systems, Ni accumulation in *T. molitor* larvae is low. Truzzi et al. (2019) reported concentrations ranging from 0.03 to 0.63 mg/kg ww, with no significant correlation between feed composition (organic wheatmeal, wheat flour, and olive‐processing by‐products) and larval Ni levels. The BAF ranged from 0.17 to 1.18 (dw), indicating negligible bioaccumulation. Although no regulatory limits exist for Ni in food, these findings suggest that Ni exposure associated with the consumption of larvae reared on the tested substrates is likely to be limited, highlighting the importance of feed selection in insect farming.

Poma et al. (2017) reported Ni in all insect species analyzed (*G. mellonella*, *L. migratoria*, *T. molitor*, and *A. diaperinus*) as well as in insect‐based products commercialized in Belgium. Concentrations remained below 0.28 mg/kg ww in all cases. In unprocessed insects, Ni content reflected limited bioaccumulation, while in processed products such as “bugballs” and croquettes, lower levels were attributed to the reduced proportion of insects (≈15.8%) and the inclusion of ingredients naturally low in Ni. These findings highlight that both substrate composition and product formulation influence Ni content, although the observed levels are low and suggest minimal risk for human consumption.

In contrast to controlled farming systems, environmental contamination can substantially influence Ni accumulation in edible insects. Mwelwa et al. (2023) investigated Ni biotransfer along the soil–plant–edible insect–human food chain in a mining‐impacted region of Zambia. Caterpillars, termites, grasshoppers, and other locally consumed insects were sampled across a 60 km gradient from the mining site. As observed for Cd, soil Ni concentrations were highest near the mining activities (31.45 µg/mg at 1.5 km) and gradually decreased with distance, reaching 13.51 µg/mg at 61.5 km, a trend that was reflected in insect samples. These findings highlight that both environmental contamination and habitat significantly influence Ni accumulation in edible insects, which is relevant for assessing potential human exposure in populations relying on insects from contaminated areas.

Murefu et al. (2023) analyzed mopane caterpillars (*G. belina*) collected from the Gwanda District in Zimbabwe and investigated the effectiveness of post‐harvest practices in reducing heavy metal contamination, particularly Ni. The post‐harvest practice categories used in this study included unprocessed samples (ungutted and naturally degutted), processed samples (manually degutted, charcoal roasted, and sun‐dried), and cooked samples (boiled and salted; boiled, salted, and roasted; and boiled, salted and fried). Ni was below the analytical detection limit in all analyzed mopane caterpillar samples, indicating either very low concentrations or its absence.

In another study, Kosečková et al. (2022) analyzed a total of 14 samples, including commercially available cricket powder (*n* = 3) and farmed insect species: house cricket, *A. domesticus* adults (*n* = 3); yellow mealworm, *T. molitor* larvae (*n* = 3); desert locust, *S. gregaria* adults (*n* = 2); and superworm, *Z. morio* larvae (*n* = 3). Samples were purchased from retail SMs and pet shops in the South Moravian region. Ni levels in edible insect samples (mg/100 g dw) were reported as follows: cricket powder (made from *A. domesticus*) ranged from 0.019 to 0.064 mg/100 g, house cricket (*A. domesticus*) from 0.05 to 0.061 mg/100 g, mealworm (*T. molitor*) from 0.046 to 0.077 mg/100 g, desert locust (*S. gregaria*) from 0.026 to 0.103 mg/100 g, and superworm (*Z. morio*) from 0.028 to 0.151 mg/100 g. Ni levels were found to be low, and the contents in the insect species remained below the applicable regulatory limits. The median Ni content was 0.0278 mg/100 g. Product (based on buffalo worm) contained the highest Ni concentration. Considering a daily consumption of 100 g, the TDI for Ni was not exceeded by any food product.

Across the included studies, Ni concentrations in edible insects were generally low under controlled farming conditions and in commercially available insect products. Variability was primarily associated with product formulation, environmental contamination, habitat characteristics, and, to a lesser extent, insect species, rather than with bioaccumulation during farming. Despite higher concentrations reported in some commercial products and mining‐impacted environments, estimated dietary exposure generally remained below established toxicological reference values, indicating limited dietary exposure under typical consumption scenarios (Poma et al. 2017; Truzzi et al. 2019; Kosečková et al. 2022; Mwelwa et al. 2023; Murefu et al. 2023; Sikora et al. 2023; Gori et al. 2025).

#### 3.1.3 Lead

Pb contamination was investigated across commercially available insect products, farmed insects, controlled feeding experiments, and environmentally exposed wild populations from Europe, Asia, Africa, and North America. Although Pb concentrations vary according to the production system, environmental contamination, processing practices, and insect species, the available evidence consistently identifies environmental exposure and rearing conditions as the primary determinants of Pb occurrence and associated dietary risk (Poma et al. 2017; Truzzi et al. 2019; Köhler et al. 2019; Van Der Fels‐Klerx et al. 2020; Kolakowski et al. 2021; Kosečková et al. 2022; Muhammad et al. 2022; Badanaro et al. 2022; Sikora et al. 2023; Murefu et al. 2023; Mwelwa et al. 2023; Holowaty et al. 2024; Gori et al. 2025).

In the European market, Gori et al. (2025) analyzed 52 insect‐based products and found that those containing more than 50% insect powder exhibited higher Pb levels (up to 0.33 mg/kg) compared with products of lower insect content (0.07 mg/kg), suggesting a direct effect of insect powder concentration on metal accumulation. Among those, 10 products were composed entirely of insect material (powder or whole), and Pb concentrations were compared with species‐specific tolerable limits established by CIRs: 2023/58 (European Commission 2023a) (≤ 0.1 mg/kg) for *A. diaperinus*, (EU) 2022/188 (European Commission 2022b) (≤ 0.05 mg/kg) and (EU) 2023/5 (European Commission 2023b) (≤ 0.1 mg/kg) for *A. domesticus*, (EU) 2021/1975 (European Commission 2021a) (≤ 0.07 mg/kg) for *L. migratoria*, (EU) 2021/882 (European Commission 2021b) (≤ 0.075 mg/kg), and (EU) 2022/169 (European Commission 2022a) (≤ 0.01/≤ 0.075 mg/kg) for *T. molitor*. The results indicated that 70% of these products exceeded these regulatory thresholds, including four *A. domesticus* samples (0.061–0.192 mg/kg), two *T. molitor* samples (0.137–0.202 mg/kg), and one *L. migratoria* sample (0.328 mg/kg), while *A. diaperinus* remained below the limit. In addition, half of these samples surpassed the 0.1 mg/kg Pb limit established by Commission Regulation (EU) 2023/915 (European Commission 2023c) (contaminants) for conventional meats (beef, lamb, pork, and poultry) (European Commission 2023c), with concentrations ranging from 0.137 to 0.320 mg/kg. Nevertheless, all remained below the higher limits for crustaceans (0.50 mg/kg) and bivalves (1.50 mg/kg) (European Commission 2023c). Notably, no statistically significant interspecies differences were observed, suggesting that Pb contamination is influenced more by rearing and processing practices than by species‐specific factors.

In contrast, Poma et al. (2017) reported consistently low Pb levels (< 0.03 mg/kg) in Belgium across four insect species (*G. mellonella*, *L. migratoria*, *T. molitor*, and *A. diaperinus*) and derived products, including locust‐ and buffalo worm‐based “bugballs,” cricket croquettes, and a “bugburger.” These concentrations were well below the regulatory benchmark value established for comparable foodstuffs (0.1 mg/kg; European Commission 2006), which was used as a comparative reference value (European Commission 2006). Similarly, Kolakowski et al. (2021) evaluated 19 samples of crickets and silkworm pupae in Canada, including dried whole insects, powders, and insect‐based products such as protein bars. Pb was detected in 58% of the samples, with detection rates higher in crickets (67%) than in silkworm pupae (25%). Concentrations ranged from 0.019 to 0.059 mg/kg (mean value of 0.034 mg/kg), with cricket‐based protein bars exhibiting the highest concentrations (0.042–0.059 mg/kg, mean 0.052 mg/kg), followed by whole insects and flours with lower yet detectable levels, while powdered cricket samples contained no detectable Pb. Silkworm pupae presented relatively low levels (∼0.020 mg/kg). All measured values were well below the Canadian regulatory benchmark value established for commonly consumed foods (0.2 mg/kg), which was used as a comparative reference value (Canada 2005a). Kitahara et al. (2022) extended this evidence to 14 commercially available insect species in Japan, including beetles, crickets, silkworms, caterpillars, and aquatic insects. Pb was detected in 64% of the samples, although several values were below the method detection limit (0.05 ppm). Among detectable samples, concentrations reached up to 0.067 ppm in sago worms, with a mean value of approximately 0.06 ppm. Despite this relatively high detection frequency, the estimated dietary exposure was considered limited, primarily due to the limited quantities typically consumed. Variability in Pb content across species was attributed to differences in environmental exposure, feeding substrates, and processing conditions. Importantly, edible insects in Japan are not covered by formal regulatory standards, highlighting the absence of comprehensive quality control and risk assessment frameworks. Comparable low Pb levels were reported by Köhler et al. (2019) in four insect species sourced from street vendors and SMs in Bangkok, Thailand: *P. succincta* (Bombay locust), *Holotrichia* spp. (scarab beetle), *A. domesticus* (house cricket), and *B. mori* (mulberry silkworm). Pb concentrations (mg/100 g fw) were as follows: Bombay locust, 0.0092; scarab beetle, 0.0117; house cricket, 0.0155 from SHs and 0.0102 from SMs; mulberry silkworm, 0.0138 from SHs and 0.0044 from SMs. Notably, the highest concentration was detected in house crickets obtained from street vendors (0.0155 mg/100 g). In the absence of specific regulatory limits for Pb in insects intended for human consumption, these concentrations were compared with the EU maximum levels established for feed materials (0.880 mg/100 g fw) and complete feed (0.440 mg/100 g fw), as defined by Commission Regulation (EU) No. 1275/2013 (European Commission 2013). Holowaty et al. (2024) assessed commercially available dried insects in France, including *A. domesticus* (crickets), *T. molitor* (mealworms), *A. laevigata* (ants), Nepidae (water bugs), *L. migratoria* (locusts), *H. longimanus* (scorpions), and *H. albostriatum* (tarantulas). Mean Pb concentrations varied markedly among species, with the highest in tarantulas (556.6 µg/kg), followed by scorpions (146.3 µg/kg), ants (105.5 µg/kg), water bugs (82.93 µg/kg), locusts (61.8 µg/kg), crickets (41.6 µg/kg), and mealworms (20.9 µg/kg). Despite pronounced interspecific variability, calculations based on the FAO/WHO PTDI (3.57 µg/kg body weight/day) suggested that most species, particularly mealworms, crickets, and locusts, could be consumed in large quantities without exceeding safety limits. Soren et al. (2021) analyzed two orthopteran species traditionally consumed by the Bodo tribe in Assam, India: *T. portentosus* and *S. monstrosus*. The specimens were collected from local markets, reflecting typical points of consumer access. Pb concentrations were significantly higher in *T. portentosus* (0.038 ± 0.001 ppm) than in *S. monstrosus* (0.008 ± 0.001 ppm). This finding suggests that Pb accumulation may reflect species‐specific biological characteristics in addition to local environmental conditions. In particular, variations in feeding behavior, ecological niche, or bioaccumulation capacity may explain the observed variation.

Controlled farmed studies support these observations. Sikora et al. (2023) analyzed 14 insect‐based food and feed products across the EU, including *A. domesticus*, *A. diaperinus*, *T. molitor*, *L. migratoria*, and *B. mori*. The median Pb concentration was 0.016 mg/100 g, with a single feed product based on *T. molitor* reaching 10.3 mg/100 g. All other food products remained well below the maximum allowances established by the EU for comparable food categories (0.1 mg/100 g dw for meat) (European Commission 2021c). Kosečková et al. (2022) analyzed the farmed insects *A. domesticus*, *T. molitor*, *Z. morio*, and *S. gregaria*. Fourteen samples were analyzed, with Pb levels ranging from 0.002 to 0.019 mg/100 g (dw). Species‐specific ranges were as follows: *S. gregaria* 0.010–0.015 mg/100 g, *A. domesticus* 0.010–0.014 mg/100 g, cricket powder 0.006–0.019 mg/100 g, *T. molitor* 0.004–0.010 mg/100 g, and *Z. morio* 0.002–0.004 mg/100 g. All values were well below the regulatory benchmark values established for crustaceans (0.05 mg/100 g) under Commission Regulation (EU) No. 1881/2006 (European Commission 2006) and for cereals (0.02 mg/100 g) as reported by EFSA, which were used as comparative reference values. In this regard, Machado et al. (2024) reported mean Pb concentrations of 0.0105 mg/kg in *T. molitor*, 0.0586 mg/kg in *L. migratoria*, and 0.0779 mg/kg in *A. domesticus*, all below the regulatory benchmark values established for comparable foodstuffs and used as comparative references under Commission Regulation (EU) No. 1881/2006 (European Commission 2006). A statistically significant difference between *T. molitor* and *A. domesticus* highlights interspecific variability, likely influenced by intrinsic factors (species‐specific metabolism and physiology) and extrinsic factors (environmental conditions, developmental stage, and season). Hyun et al. (2012) examined *O. chinensis formosana* under controlled rearing conditions and found that the Pb concentration in dried samples ranged from 0.12 to 0.29 mg/100 g and in fresh samples was ∼0.12 mg/100 g. Although formal regulatory limits for grasshoppers have not been established, the intake remains below that associated with conventional animal protein sources.

Controlled experiments demonstrated that diet and substrate influence Pb accumulation. Truzzi et al. (2019) examined *T. molitor* larvae reared on wheat flour or wheatmeal, either alone or supplemented with increasing proportions of organic olive pomace (25%, 50%, and 75%). Pb concentrations in substrates ranged from 0.005 to 0.039 mg/kg (12% moisture), with wheat flour containing the lowest and wheatmeal containing the highest levels, all well below the European Commission legal limit for complete animal feed of 5 mg/kg (European Commission 2002). In contrast, Pb concentrations in larvae were substantially higher, ranging from 0.19 to 0.29 mg/kg (dw), with BAFs between 5 and 34. Larvae fed exclusively wheat flour exhibited the lowest Pb accumulation, whereas those reared on substrates containing 25% wheatmeal and 75% olive pomace showed the highest levels. No significant correlation was observed between substrate and larval Pb, suggesting additional sources of contamination, such as organic carrots provided as a water source, which can bioaccumulate Pb above the EU limit for vegetables (0.1 mg/kg ww) (European Commission 2011a). Despite higher Pb levels in larvae compared with substrates, all values remained below the EU safety limit for food products of animal origin (0.1 mg/kg; 1881/2006/EC and its amendment 420/2011/EC) (European Commission 2006, 2011a). In a diet‐dependent study, Kim et al. (2020) investigated Pb concentrations in *L. migratoria* from South Korea farms, comparing individuals fed corn and wheat. Pb levels differed markedly between feeding groups, with corn‐fed locusts exhibiting concentrations of 0.13 mg/kg, whereas wheat‐fed individuals showed substantially lower levels (0.01 mg/kg). Notably, both values fall within the Pb contamination ranges generally applied to edible insects in South Korea (0.1–0.3 mg/kg for Pb), as reported in the study. Similarly, Van Der Fels‐Klerx et al. (2020) assessed Pb in *H. illucens* larvae reared on substrates containing 3%–6% packaging residues derived from former food products. Four substrate types were evaluated: meat‐based plastic (MP), vegetarian plastic (VP), meat‐based carton (MC), and vegetarian carton (VC). Pb concentrations in larvae ranged from 0.06 mg/kg (MP) to 0.09 mg/kg (VP), whereas all substrates contained Pb below the limit of quantification (0.062 mg/kg). All measured values were substantially lower than the maximum residue limits of 5 mg/kg for feed and 10 mg/kg for larvae (European Commission 2002), indicating that Pb detected in the larvae originated from background contamination in the substrates rather than from biologically significant accumulation.

Environmental studies further highlight the influence of ecological exposure on Pb accumulation in edible insects. In this regard, Badanaro et al. (2022) evaluated 12 edible insect species from Togo, reporting Pb levels ranging from 0.041 mg/kg in *G. varians* to 0.501 mg/kg in *C. tripunctatus*, with the latter slightly exceeding the regulatory benchmark value (0.5 mg/kg) established under Commission Regulation (EC) No. 1881/2006 (European Commission 2006), which was used as a comparative reference. Terrestrial insects, such as *M. bellicosus* and *B. membranaceus*, generally exhibited lower Pb concentrations, whereas aquatic species, exemplified by *C. tripunctatus*, accumulated higher levels due to the greater bioavailability of Pb in freshwater ecosystems. These results suggest that ecological and dietary factors, including habitat and trophic interactions, play a key role in determining metal uptake in edible insects. Similarly, Muhammad et al. (2022) analyzed grasshoppers, locusts, and termites traditionally consumed in the Dutsin‐Ma region of Niger, assessing Pb levels against FAO/WHO maximum permissible limits (2 mg/kg dw) (Guidelines for the Safe Use of Wastewater, Excreta and Graywater, Volume 2, n.d.). Pb concentrations were 0.7 ± 0.58 mg/kg in grasshoppers, 0.04 ± 0.08 mg/kg in locusts, and 0.23 ± 0.006 mg/kg in termites. Although all values remained below the recommended limits, the presence of Pb confirms environmental exposure to this toxic metal. Grasshoppers exhibited the highest concentration, likely reflecting species‐specific bioaccumulation influenced by feeding behavior, ecological niche, or habitat contamination. Devkota and Schmidt (2000) analyzed four acridid grasshopper species inhabiting a mountainous grassland in the Taigetos Mountains, Greece: *Calliptamus italicus*, *Oedipoda caerulescens*, *Oedipoda germanica*, and *Chorthippus (Glyptobothrus) crassiceps*. To assess potential trophic transfer, Pb levels in grasshoppers were compared with those in their respective food plants. The measured Pb concentrations in the insects ranged from 0.32 to 0.62 mg/kg dw, with the highest concentration observed in *O. germanica* (∼0.60 mg/kg), followed by *O. caerulescens* (∼0.45 mg/kg), *C. italicus* (∼0.43 mg/kg), and *C. (Glyptobothrus) crassiceps* (∼0.43 mg/kg). In all cases, Pb concentrations in the grasshoppers were lower than those in their food plants, indicating an absence of biomagnification in these species. These findings suggest that although Pb is present in both plants and herbivorous insects, its transfer to higher trophic levels does not lead to accumulation in the studied grasshopper species.

Environmental exposure studies, such as Mwelwa et al. (2023) in Zambia, demonstrated Pb transfer along the soil–plant–insect–human food chain. Soil Pb levels were highest near the mining site (16.8 µg/mg at 1.5 km) and gradually decreased with distance, reaching 6.5 µg/mg at 61.5 km, demonstrating the strong influence of proximity to mining on soil contamination. Pb levels in host plants mirrored this gradient (3.7–9.2 µg/mg), confirming their role as conduits for metal transfer to higher trophic levels. Insect Pb accumulation was highly species specific: *Imbrasia obscura* exhibited the highest content (136.7 µg/mg), followed by *Imbrasia epimethea* (63.9 µg/mg), *Cirina forda* (47.2 µg/mg), and *Macrotermes falciger* (28.7 µg/mg). Correlations between insect and plant Pb were weaker than those between soil and plants, highlighting that bioaccumulation is largely dependent on species‐specific physiological and ecological traits. EDI calculations, based on local consumption patterns, suggested that ingestion of certain insects, particularly *Imbrasia rubra* (8350 µg/mg) and *I. obscura* (1058 µg/mg), from areas near the mining site could exceed the FAO/WHO provisional TDI for Pb (400 µg/mg) (City Region Food System Situational Analysis. Kitwe, Zambia FAO—Food for the Cities Programme, n.d.). These results demonstrate a clear trophic transfer of Pb from soil to plants and insects, with species identity and proximity to contamination sources critically shaping human exposure risk.

Processing and cooking methods also influence Pb levels. In this regard, Murefu et al. (2023) evaluated *G. belina* at different post‐harvest stages, including fresh, naturally degutted, and manually degutted samples. Pb was detected at low levels in fresh ungutted samples (0.003 mg/kg), manually degutted caterpillars (0.004 mg/kg), and naturally degutted specimens (0.002 mg/kg). In contrast, all processed and cooked samples contained Pb below the limit of detection. Importantly, Pb concentrations in fresh samples remained well below the regulatory benchmark values used as comparative references, demonstrating that post‐harvest processing and cooking effectively reduce potential dietary exposure to metal contaminants. Similarly, Marzoli et al. (2023) analyzed *B. mori* and its derived products, including chrysalises, chrysalis oil, and defatted chrysalis meal. Pb was detected in all samples, ranging from 0.021 to 0.45 mg/kg, with the highest concentration observed in defatted chrysalis meal, highlighting that processing can modulate heavy metal content in silkworm‐derived products. Zhang et al. (2009) compared Pb concentrations with those of soil to reveal their biogeochemical processes along food chains in Huludao City, Liaoning Province, China. Seven arthropod taxa, including *L. migratori manilensis*, *Acrida chinensis*, *Holotrichia* spp., *Eligma narcissus* larvae, mantises, spiders, and dragonflies, were collected by hand. The CFs for Pb in the soil–plant–herbivorous insect–carnivorous insect food chain were 1.47, 2.24, and 0.57 mg/kg, respectively. Pb did not biomagnify as expected toward higher trophic levels, as its accumulation in carnivorous insects was not pronounced. Among the four herbivorous insect species examined, the highest metal concentrations were found in *E. narcissus* larvae, while the lowest were observed in *L. migratori manilensis*. The average Pb concentration in plants was 325.3 mg/kg, showing significant variation among plant species. These differences were attributed to variations in the distance from pollution sources and differences in metal uptake capacities. In the present study, plant Pb concentrations were higher than those in soil, with a CF of 1.47, indicating that Pb accumulates in plants.

Studies included in this review indicate that Pb concentrations in edible insects were generally low under controlled farming conditions and in most commercially available products. Variability was primarily associated with environmental contamination, rearing substrate, processing practices, and habitat characteristics rather than with consistent species‐specific bioaccumulation. Although elevated Pb concentrations were reported in insects collected from mining‐impacted areas and in a limited number of commercial products, exposure assessments generally indicated a low dietary risk under typical consumption scenarios while emphasizing the importance of environmental monitoring and quality control throughout the production chain (Poma et al. 2017; Truzzi et al. 2019; Van Der Fels‐Klerx et al. 2020; Kolakowski et al. 2021; Kosečková et al. 2022; Muhammad et al. 2022; Badanaro et al. 2022; Sikora et al. 2023; Murefu et al. 2023; Mwelwa et al. 2023; Holowaty et al. 2024; Gori et al. 2025).

#### 3.1.4 Mercury

Hg concentrations were investigated in commercially available insect products, farmed insects, controlled feeding experiments, and environmentally exposed wild populations from Europe, Asia, and Africa. Despite differences in insect species, production systems, environmental conditions, and analytical approaches, the available studies consistently reported low Hg concentrations under controlled farming conditions, while higher levels were primarily associated with environmental contamination and trophic transfer in wild ecosystems (Köhler et al. 2019; Truzzi et al. 2019; Van Der Fels‐Klerx et al. 2020; Kolakowski et al. 2021; Badanaro et al. 2022; Kitahara et al. 2022; Sikora et al. 2023).

Badanaro et al. (2022) evaluated Hg levels in 12 edible insect species from Togo, including *C. tripunctatus*, *G. trivittata*, *G. impressa*, *G. varians*, *R. sobrina*, *P. cordata*, *P. marginata*, *S. interrupta*, *M. bellicosus*, *A. ruficornis*, *K. angulífera*, and *B. membranaceus*. Hg concentrations ranged from 0.010 mg/kg in *G. varians* to 0.315 mg/kg in *G. trivittata*, with all values remaining below the EU maximum limit for Hg in food (0.5 mg/kg for Hg) (European Commission 2006). Similarly, Köhler et al. (2019) reported very low Hg levels in four insect species (*P. succincta*, *Holotrichia* spp., *A. domesticus*, and *B. mori*) collected from street vendors and SMs in Bangkok, Thailand. The highest Hg concentration was found in *Holotrichia* spp. (0.008 mg/100 g fw), while all other species exhibited levels below 0.005 mg/100 g fw. These values were well below EU regulatory thresholds for feed materials (0.009 mg/100 g fw; Directive 2002/31/EC) (European Commission 2002). In Japan, Kitahara et al. (2022) detected Hg in 29% of 14 commercially available insect species (including beetles, crickets, silkworms, sago worms, and scorpions), with concentrations ranging from below the detection limit to 0.50 ppm (highest in a June beetle). Despite this detection frequency, exposure was considered low due to the small quantities typically consumed.

Retail‐based assessments further support these findings. Kolakowski et al. (2021) analyzed 19 retail insect products, including crickets and silkworm pupae, in Canada and detected Hg in 74% of the samples, with concentrations ranging from 0.00094 to 0.028 mg/kg (mean: 0.0062 mg/kg). Crickets exhibited higher Hg content than silkworm pupae, with powdered cricket samples reaching the highest concentration (0.028 mg/kg), followed by whole insects and protein bars. In contrast, cricket flour contained the lowest detectable levels (0.001–0.0011 mg/kg), while Hg was undetectable in all silkworm pupae. All values were below the Canadian maximum limit for Hg in foods (0.5 mg/kg) (Canada 2005b), indicating compliance with the applicable regulatory benchmark for Hg concentrations. Differences in Hg content among cricket products likely reflect species‐specific accumulation, product type, processing methods, developmental stage, and environmental conditions during cultivation or harvest. Hg was undetectable in all silkworm pupae. All quantified concentrations were below the Canadian maximum limit for Hg in foods (0.5 mg/kg) (Canada Health 2005b), indicating compliance with this regulatory comparator. Differences in Hg content among cricket products likely reflect species‐specific accumulation, product type, processing methods, developmental stage, and environmental conditions during cultivation or harvest. Likewise, Sikora et al. (2023) reported Hg levels below the detection limit (< 0.015 mg/100 g) in both insect‐based food and feed products, including *A. domesticus*, *T. molitor*, and *B. mori*. These concentrations are substantially below the maximum allowable limit for food supplements (0.1 mg/100 g dw) established by the European Commission (European Commission 2006 and 2002/617), indicating negligible Hg contamination.

Under controlled rearing conditions, Hg accumulation appears to be limited and only weakly influenced by diet. Hyun et al. (2012) reported extremely low Hg concentrations in *O. chinensis formosana* (0.003–0.007 mg/100 g), confirming minimal bioaccumulation in insects reared in clean environments. Similarly, Kim et al. (2020) observed consistently low Hg levels (0.01 mg/kg) in *L. migratori* reared on both corn‐ and wheat‐based diets, indicating that Hg uptake in this species is not markedly affected by feed composition.

Controlled feeding studies further support these findings. Truzzi et al. (2019) evaluated Hg transfer in *T. molitor* larvae reared on wheat‐based diets, either alone or supplemented with increasing proportions of organic olive pomace. Hg concentrations in all feeding substrates were extremely low, not exceeding 1 µg/kg dw, and were markedly below the maximum limit established by the European Commission for undesirable substances in animal feed (0.1 mg/kg at 12% moisture; European Commission 2002). Among the substrates, organic olive pomace exhibited relatively higher Hg concentrations than wheat‐based feeds, likely reflecting the intrinsic metal content of plant‐derived residues used in feed formulation. Hg concentrations in larvae ranged from 0.3 to 1.6 µg/kg dw and showed a strong positive linear correlation with substrate Hg content, confirming bioaccumulation in *T. molitor*, with BAF varying between 1.5 and 6.9. Despite this transfer, Hg levels in both substrates and larvae remained well below regulatory limits established for food products of animal origin (European Commission 2011a), supporting the safety of these feeding strategies for insect rearing. Van Der Fels‐Klerx et al. (2020) assessed Hg occurrence and bioaccumulation in *H. illucens* larvae reared on four waste‐based substrates (meat‐based or vegetarian, combined with plastic or cardboard). Hg concentrations in both substrates and larvae were below the analytical limit of quantification (LOQ: 0.02 mg/kg). Correspondingly, BAFs were close to zero, indicating negligible uptake of Hg regardless of substrate composition or production system. All measured values were substantially below the maximum levels established by the EU (0.1 mg/kg for both feed materials and larvae; European Commission 2002), confirming the absence of relevant Hg accumulation even under conditions involving waste‐derived feed substrates.

In contrast to these generally low levels, higher Hg concentrations have been reported in wild insects from specific environments. Devkota and Schmidt (2000) reported Hg levels ranging from 0.70 to 2.04 mg/kg dw in four acridi grasshopper species *(C. italicus*, *O. caerulescens*, *O. germanica*, and *C. (Glyptobothrus) crassiceps*) collected from mountainous grasslands in Greece. The highest levels were observed in *C. (Glyptobothrus) crassiceps* (∼2.042 mg/kg), followed by *O. caerulescens* (∼0.96 mg/kg), *C. italicus* (∼0.92 mg/kg), and *O. germanica* (∼0.71 mg/kg). In several cases, Hg levels in several species were comparable to or exceeded those in their dietary plants, suggesting a greater potential for trophic transfer and bioaccumulation in terrestrial herbivorous insects.

In the research by Zhang et al. (2009), the Hg concentrations of ached plants and insect samples were investigated and compared with those of soil to reveal their biogeochemical processes along food chains in Huludao City, Liaoning Province, China. Seven insect species, *L. migratori manilensis*, *A. chinensis*, *Holotrichia*, *E. narcissus* larvas, mantis, spider, and dragonfly, were collected by hand. Hg, the CFs across the soil–plant–herbivorous insect–carnivorous insect food chain were 0.18, 6.57, and 7.88 mg/kg, respectively. In the soil–plant–insect system, Hg concentrations in soil were above local background levels and reached 73 times the crustal Clarke values, indicating severe contamination. In plants, the average Hg concentration was 1.1 mg/kg, with a CF of 0.18, indicating no significant accumulation from soil to plants. In foliage, the average Hg concentration was 0.99 mg/kg, while strong biomagnification was observed in insects: in *E. narcissus* larvae, the Hg CF reached 13.09, and in the *Ulmus pumila*–*Holotrichia* food chain, Hg was enriched 4.71‐fold. Among carnivorous insects, the highest Hg concentrations were found in dragonflies, indicating continued transfer and accumulation of Hg at higher trophic levels. Overall, Hg was the most strongly biomagnified element, with noticeable accumulation observed in carnivorous insects as the food chain extended to secondary consumers. The results indicated that CFs depended on both the type of metal and the insect species within the food chains.

Current evidence indicates that Hg concentrations in edible insects are generally very low under controlled farming conditions and in commercially available insect products. Variability was mainly associated with environmental contamination and trophic position rather than with farming practices or diet composition. Although elevated Hg concentrations were reported in wild insects collected from contaminated ecosystems, studies conducted under commercial and experimental production conditions consistently indicated low consumer exposure, supporting the safety of farmed edible insects with respect to Hg contamination (Truzzi et al. 2019; Van Der Fels‐Klerx et al. 2020; Kolakowski et al. 2021; Badanaro et al. 2022; Kitahara et al. 2022; Sikora et al. 2023; Devkota and Schmidt 2000; Z.‐S. Zhang et al. 2009).

### 3.2 Acrylamide and PAHs

#### 3.2.1 PAHs

PAHs have been investigated primarily under controlled rearing conditions to evaluate the influence of production substrates on contaminant transfer to edible insects. Although the available evidence is limited, current studies consistently indicate low PAH accumulation in farmed insects, with contamination largely determined by substrate characteristics rather than by intrinsic species‐specific bioaccumulation (Charlton et al. 2015; Van Der Fels‐Klerx et al. 2020).

Charlton et al. (2015) analyzed PAH contamination in larvae of four fly species, *M. domestica*, *C. vomitoria*, *Chrysomya* spp., and *H. illucens*, reared on various waste substrates across different geographic locations, including the United Kingdom, China, Mali, and Ghana. Larval PAH4 (sum of benzo[*a*]pyrene, benz[*a*]anthracene, benzo[*b*]fluoranthene, and chrysene) concentrations ranged from 0.28 to 9.82 µg/kg.

Although EU regulatory limits for PAHs in animal feed are not established, food‐based thresholds range from 1 µg/kg in baby foods to 35 µg/kg in smoked seafood (EC regulation 1881/2006 as amended by 835/2011) (European Commission 2006, 2011b). Some larvae exceeded the lower thresholds, indicating potential safety concerns. These findings suggest that PAH contamination is influenced by insect species, rearing substrate, and geographic origin, emphasizing the need for standardized monitoring protocols to ensure the safety of insects intended for feed or food applications.

Similarly, Van Der Fels‐Klerx et al. (2020) assessed PAH accumulation in *H. illucens* larvae reared on former food products containing shredded packaging material. Four substrate types were tested: meat‐based plastic packaging, vegetarian plastic packaging, meat‐based carton packaging, and vegetarian carton packaging, each containing 3%–6% packaging residues. The sum of 16 priority PAHs (∑PAH_16_) in larvae was ≤ 0.002 mg/kg across all treatments, whereas substrate concentrations ranged between 0.002 and 0.007 mg/kg. BAFs varied from 0.5 to 1.2 g substrate/g organism, indicating negligible accumulation of PAHs in larvae, regardless of substrate composition. No maximum residue limits have been established for PAHs in feed products within the EU. For food products, Regulation (EC) No 1881/2006 (European Commission 2006) sets legal thresholds for ∑PAH_4_, used as markers for ∑PAH_16_, ranging from 1.0 µg/kg in infant formulae to 50.0 µg/kg in dried herbs and spices (European Commission 2006). The concentrations detected in *H. illucens* larvae were substantially below these limits, indicating minimal risk of PAH transfer from the substrates under the tested rearing conditions.

Findings from the available studies suggest that PAH concentrations in edible insects are generally low under controlled farming conditions. Variability appears to be primarily influenced by substrate composition and rearing conditions, while bioaccumulation in insect tissues remains limited. Although isolated exceedances of the most stringent food‐based comparator values were reported, the available findings indicate limited transfer of PAHs to edible insects when appropriate production practices are applied (Charlton et al. 2015; Van Der Fels‐Klerx et al. 2020).

#### 3.2.2 Acrylamide

Available studies on acrylamide have focused on insect‐enriched bakery products to evaluate whether the incorporation of edible insects influences acrylamide formation during thermal processing. Despite differences in insect species, supplementation levels, processing conditions, and formulation strategies, the findings consistently indicate that insect incorporation does not increase acrylamide concentrations beyond regulatory benchmark levels and that processing interventions, particularly fermentation, may further mitigate acrylamide formation (Burešová et al. 2023; Bartkiene et al. 2023; Jankauskienė et al. 2024).

Burešová et al. (2023) investigated the effect of incorporating *A. domesticus* (cricket) and *T. molitor* (yellow mealworm) powders on acrylamide formation in wheat bread, both leavened and unleavened, at supplementation levels of 5%, 8%, and 12%. The addition of insect powders increased the content of reducing sugars and free amino acids in the raw materials; however, asparagine, the limiting precursor for acrylamide formation in cereal products, was reduced in the most enriched samples (242.4 mg/kg for crickets and 177.9 mg/kg for mealworms) compared to control wheat flour (249.6 mg/kg). In unleavened bread, acrylamide levels increased slightly in the samples with the highest insect supplementation (14.03 µg/kg for crickets and 29.52 µg/kg for mealworms vs. 11.02 µg/kg for the control group), whereas in leavened bread, the most enriched variants showed lower acrylamide concentrations than the control (64.84 and 68.78 µg/kg vs. 82.47 µg/kg). These findings indicate that insect supplementation enhances the nutritional profile of bakery products without increasing the risk of acrylamide exposure for consumers.

Similarly, Jankauskienė et al. (2024) evaluated wheat bread enriched with frozen *T. molitor* powder at 5%, 10%, and 15% and baked at 220°C for 25 min. Acrylamide levels were analyzed, considering the maximum threshold of 50 µg/kg established by Commission Regulation (EU) No. 2017/2158 (European Commission 2017b). All samples, including those enriched with 15% mealworm powder, exhibited acrylamide concentrations well below this limit, ranging from 21.2 ± 0.02 to 30.4 ± 0.01 µg/kg. No consistent statistical trend was observed between insect concentration and acrylamide formation; however, the highest values were found in bread containing 15% larvae reared on spent grain from beer production. These findings indicate that incorporating yellow mealworm powder into wheat bread does not result in acrylamide concentrations exceeding regulatory limits, supporting its compliance with the applicable regulatory benchmark for acrylamide while highlighting its potential as a sustainable protein source.

The influence of fermentation on acrylamide mitigation in insect‐enriched bread was explored by Bartkiene et al. (2023). Wheat bread was supplemented with *A. domesticus* at 10%, 20%, and 30%, either untreated or fermented with *Lactiplantibacillus plantarum* No. 122. Although cricket flour contributes additional acrylamide precursors, fermentation effectively reduced acrylamide formation in breads containing 10% and 20% cricket flour, with reductions of 33.2% and 31.7%, respectively, compared to breads with untreated flour. No significant differences in acrylamide content were observed in breads containing 30% cricket flour, which showed average levels of 42.7 µg/kg. The addition of cricket flour also affected crust color, likely due to an intensified Maillard reaction, and contributed to higher aroma compound formation. These findings demonstrate that fermentation is an effective strategy to reduce acrylamide formation in insect‐enriched bakery products, particularly at lower levels of insect supplementation, while maintaining product quality.

The findings indicate that incorporating edible insect ingredients into bakery products does not compromise acrylamide safety under the evaluated processing conditions. Acrylamide formation appears to be influenced primarily by formulation and processing parameters, including insect inclusion level and fermentation, rather than by the presence of insect‐derived ingredients alone. The available evidence therefore supports the use of edible insects in bakery products without increasing acrylamide exposure while highlighting fermentation as a promising strategy to further reduce contaminant formation (Burešová et al. 2023; Bartkiene et al. 2023; Jankauskienė et al. 2024).

## 4 Discussion

Throughout this review, contaminant concentrations are interpreted in relation to available regulatory benchmark values and toxicological reference points where available in the original studies. These comparisons should not be interpreted as formal quantitative risk assessments, as actual consumer risk depends on dietary exposure, consumption patterns, and population‐specific factors. The heterogeneity in exposure assumptions and toxicological reference values across studies limits direct comparability and highlights the lack of standardized methodologies for assessing insects as food. This variability underscores the urgent need for harmonized exposure assessment methodologies and insect‐specific consumption data to support future regulatory risk assessment frameworks.

### 4.1 Critical Appraisal of Heavy Metal Risk Interpretation in Insect‐Based Edible Products

Gori et al. (2025) assessed Cd concentrations in insect‐based products commercially available in the EU and reported that 10% of samples composed entirely of insect material exceeded the tolerable Cd intake. While this study provides valuable occurrence data for novel food matrices, the interpretation of its results raises important methodological and risk assessment considerations.

First, only a subset of the analyzed products, those consisting exclusively of insect powder or whole insects, was compared with regulatory limits established under specific CIRs. This selective comparison limits the broader applicability of the findings, particularly given that composite products may also contribute to dietary exposure.

Second, the use of product‐level concentration data without a clearly defined consumption scenario complicates the interpretation of exceedance in relation to tolerable intake values. Tolerable intake values, such as TDI or TWI, are population‐based health guidance metrics that require the integration of both concentration data and realistic consumption patterns rather than being inferred directly from analytical results alone.

In contrast, studies such as Truzzi et al. (2019) provide a more comprehensive exposure‐oriented framework. In their assessment of Ni concentrations in *T. molitor* larvae, the authors not only reported occurrence data but also evaluated BAF and estimated dietary exposure based on realistic consumption scenarios. Despite the absence of maximum levels for Ni in food under current EU legislation, the comparison with EFSA‐established health‐based guidance values (TDI and acute reference dose [ARfD]) demonstrated that estimated dietary exposure remained well below levels of concern for the general population. This approach allows for a more nuanced interpretation of risk, distinguishing between contaminant presence and actual health relevance.

Applied to the findings of Gori et al. (2025), a similar exposure‐based assessment would be necessary to substantiate conclusions regarding Cd‐related risk. Without explicit assumptions regarding portion size, consumption frequency, body weight, and cumulative dietary exposure, statements referring to exceedance of tolerable intake may overestimate potential risk. This is particularly relevant for insect‐based foods, which currently represent niche products with relatively low consumption levels in most European populations.

For Cd, EFSA established a TWI of 2.5 µg/kg body weight per week, a value confirmed as protective of human health. This TWI represents the appropriate benchmark for risk characterization and should be used to evaluate whether estimated dietary exposure from insect consumption contributes meaningfully to overall Cd intake.

Without such an exposure‐based comparison, statements referring solely to exceedance of specification limits risk overstating potential health concerns. Given the currently low consumption levels of insect‐based foods in most European populations and the absence of specific MLs under Regulation (EU) 2023/915 (European Commission 2023c) (contaminants), an EFSA‐aligned approach requires moving beyond compliance checks toward integrated exposure assessment.

Overall, these considerations highlight the importance of integrating occurrence data with consumption scenarios and toxicological reference values when assessing chemical risks in novel foods. The comparison between Cd and Ni studies underscores that exceedance of concentration‐based thresholds does not automatically translate into meaningful health risk, reinforcing the need for harmonized, EFSA‐aligned exposure assessment approaches in the evaluation of contaminants in entomophagy.

Concerning Pb, Poma et al. (2017) investigated Pb contamination in four insect species and related insect‐based food products commercialized in Belgium and reported consistently low concentrations, with all values remaining below 0.03 mg/kg fw. When compared with established European maximum levels for comparable foodstuffs, these concentrations were substantially lower than the applicable regulatory thresholds.

The authors concluded that consumption of the analyzed insect species did not pose additional heavy metal‐related health risks compared with conventional animal protein sources. This exposure‐compatible interpretation aligns with an EFSA‐oriented framework, in which occurrence data are contextualized against established toxicological benchmarks rather than interpreted in isolation.

Nevertheless, other investigations have demonstrated that Pb concentrations may vary depending on the product format, environmental context, and rearing conditions. Gori et al. (2025) reported higher Pb levels in powdered products containing high proportions of insect material, with several samples exceeding species‐specific limits defined under CIRs.

Similarly, field‐based studies conducted in environmentally impacted regions, such as those by Devkota and Schmidt (2000) and Mwelwa et al. (2023), documented elevated Pb concentrations associated with soil contamination gradients. However, these findings reflect context‐dependent environmental exposure rather than systematic bioaccumulation under controlled production systems.

Evidence from regulated farming environments further supports this distinction. Studies evaluating farmed species under controlled substrates, including Van Der Fels‐Klerx et al. (2020), have consistently reported Pb concentrations below applicable regulatory thresholds, indicating that contamination is primarily driven by external environmental and substrate factors. Collectively, these findings reinforce that Pb risk characterization in entomophagy should be grounded in integrated exposure assessment and production‐chain control rather than inferred solely from isolated concentration exceedances.

Concerning Hg, Badanaro et al. (2022) evaluated Hg concentrations in twelve insect species commercialized for human consumption in Togo and reported generally low levels, ranging from 0.010 to 0.315 mg/kg, with all values remaining below the regulatory benchmark values used as comparative references for Hg in food. The authors concluded that the observed concentrations represent minimal risk of Hg exposure through insect consumption. This interpretation reflects an exposure‐compatible perspective, in which measured concentrations are contextualized against established regulatory benchmarks rather than considered inherently hazardous.

Nevertheless, variability in Hg occurrence has been documented under specific environmental conditions. Devkota and Schmidt (2000) reported higher Hg concentrations in wild‐collected grasshopper species from a mountainous region in Greece, with evidence suggesting potential trophic transfer from contaminated vegetation. However, these findings reflect localized environmental contamination rather than systematic accumulation within regulated production systems.

Evidence from controlled rearing studies further supports this distinction. Investigations conducted under standardized feeding conditions, including Truzzi et al. (2019) and Van Der Fels‐Klerx et al. (2020), have consistently reported extremely low or nondetectable Hg concentrations in both substrates and larvae, with values well below established maximum residue limits. Collectively, these data indicate that under controlled farming conditions, Hg does not accumulate to levels of toxicological concern.

Accordingly, meaningful risk characterization should integrate realistic consumption patterns and toxicological reference values rather than infer health relevance solely from the detection of trace concentrations.

An additional point requiring clarification concerns the interpretation of exceedances relative to CIRs. These regulations authorize specific insect species as novel foods and define product specifications, including maximum levels for certain contaminants expressed as concentration limits in the food matrix (mg/kg). Importantly, these specification limits are not toxicological reference values and should not be interpreted as indicators of health‐based intake thresholds. Rather, they serve as compliance criteria to ensure product quality and consistency at the point of authorization.

Consequently, statements referring to products that “exceeded the maximum level established by CIRs” do not, by themselves, imply a health risk, unless such exceedances are contextualized within an exposure assessment framework that considers consumption patterns and established toxicological reference values, such as TDIs or benchmark dose levels.

To provide a structured overview of the toxicological reference values, regulatory benchmarks, and risk assessment approaches discussed across metals, PAHs, and acrylamide, Table 2 summarizes the main frameworks applied in the evaluated studies. This synthesis highlights the distinction between concentration‐based compliance criteria and exposure‐based risk characterization, reinforcing the importance of integrating toxicological guidance values with realistic consumption scenarios when interpreting contaminant occurrence in insect‐derived products intended for human consumption and within the broader context of entomophagy.

**TABLE 2:** Comparative overview of toxicological benchmarks and exposure‐based risk interpretation.

Contaminant	Toxicological reference	Comparative regulatory benchmark	RA performed	Key risk interpretation
Cd	TWI (EFSA)	Reg. (EU) 2023/915	Yes	Estimated dietary exposure generally represented a low proportion of the TWI; potential risk remained exposure dependent
Pb	BMDL (EFSA)	Reg. (EU) 2023/915	Limited	Some reported concentrations exceeded comparative regulatory benchmark values under specific scenarios; interpretation depends on estimated dietary exposure
Hg	TWI (EFSA)	Reg. (EU) 2023/915	Compliance‐based	Measured concentrations generally remained below comparative regulatory benchmark values, with limited contaminant accumulation reported in farming systems
Ni	TDI (EFSA)	No EU maximum levels	Yes	Estimated dietary exposure generally remained below the TDI, indicating limited bioaccumulation under the reported conditions
PAHs	MOE (EFSA)	Reg. (EU) 2023/915	Partial	Contaminant accumulation was influenced by substrate composition and exposure conditions; formal exposure assessment was limited
Acrylamide	MOE (EFSA)	Reg. (EU) 2017/2158	No	Formation was processing dependent, with reported concentrations generally remaining below comparative benchmark values

*Note*: Comparative regulatory benchmark values are presented for contextual interpretation of contaminant occurrence and should not be interpreted as insect‐specific maximum limits or as formal quantitative risk assessments.

Abbreviations: BMDL, benchmark dose lower confidence limit; EFSA, European Food Safety Authority; MOE, margin of exposure; RA, risk assessment; TDI, tolerable daily intake; TWI, tolerable weekly intake.

### 4.2 Critical Appraisal of PAH and Acrylamide Risk Interpretation in Insect‐Based Edible Products

The findings summarized in Section 3.2 highlight the relevance of process‐related contaminants, namely, PAHs and acrylamide, in the context of entomophagy as an alternative protein source. From an EFSA‐aligned risk assessment perspective, these results should be interpreted through a clear distinction between hazard presence, regulatory compliance, and dietary risk, particularly given the absence of insect‐specific contaminant limits in current European legislation.

Concerning PAHs, EU contaminant legislation establishes maximum levels for benzo[*a*]pyrene and the ∑PAH_4_ group in foodstuffs under Regulation (EU) 2023/915 (European Commission 2023c), which consolidates and updates previous provisions. However, this regulation does not define specific food categories for insects used for entomophagy, and EFSA has acknowledged that harmonized maximum levels for metals and PAHs in such products are currently lacking. Consequently, the exceedance of PAH limits reported in some experimental studies should be interpreted primarily as an indication of potential noncompliance with food‐based benchmarks rather than as direct evidence of consumer health risk.

In line with EFSA guidance, risk characterization requires the integration of occurrence data with exposure assessment, considering realistic consumption patterns and the toxicological relevance of the detected compounds. The studies discussed in Section 3.2 indicate that PAH accumulation in insects is primarily influenced by rearing substrates and the level of environmental contamination rather than by the insect species themselves. This supports EFSA's broader conclusion that control of environmental and feed composition is central to ensuring food safety, particularly for emerging food categories.

Regarding acrylamide, EFSA has consistently emphasized that this contaminant is formed during thermal processing and should be managed through process control and mitigation strategies rather than through raw material exclusion. This approach is reflected in Commission Regulation (EU) 2017/2158 (European Commission 2017b) (acrylamide mitigation), which establishes benchmark levels and mitigation measures for acrylamide in specific food categories, including bread and cereal‐based products.

The evidence summarized in Section 3.2 aligns with EFSA's assessment that ingredient composition alone does not determine acrylamide risk and that appropriate processing conditions can effectively limit consumer exposure, even when insect‐derived ingredients are incorporated.

A comparable distinction can be observed within the regulatory framework of the U.S. Food and Drug Administration (FDA), which adopts a risk management‐oriented approach based on action levels and exposure reduction strategies rather than fixed health‐based intake values such as TWI or TDI. In the context of process contaminants such as PAHs and acrylamide, the FDA emphasizes mitigation guidance and industry responsibility to minimize formation, rather than establishing contaminant‐specific maximum levels for novel food categories. This transatlantic regulatory contrast further underscores that the presence or even exceedance of food‐based benchmarks should not be interpreted in isolation from exposure assessment and toxicological context.

### 4.3 Limitations and Methodological Considerations

Despite providing a comprehensive synthesis of the available literature, this scoping review also highlights several methodological limitations in the current body of evidence on chemical contaminants in edible insects. First, studies frequently report contaminant concentrations using heterogeneous units (e.g., mg/kg ww vs. dw), which complicates direct comparisons across datasets. In addition, considerable variability is observed across insect species, rearing substrates, environmental conditions, and geographical contexts. Another important limitation relates to the frequent aggregation of results from insects produced under controlled farming systems and those harvested from wild or environmentally contaminated areas. Since exposure scenarios and contamination pathways may differ substantially between these contexts, clearer differentiation between farmed and wild‐harvested insects would strengthen future risk‐oriented analyses.

Furthermore, many studies interpret contaminant concentrations in insects by comparison with regulatory limits established for other food categories (e.g., meat, fish, cereals, or feed). This reflects the current regulatory gap, as insect‐specific maximum levels for most contaminants are not yet established within the European regulatory framework. More broadly, the reviewed literature indicates that most studies report occurrence data rather than performing full dietary exposure or risk assessments. While measured contaminant concentrations are often compared with regulatory limits, such comparisons alone do not allow conclusions about actual health risks. The potential risk associated with contaminants depends not only on their concentration in food but also on the amount and frequency of consumption.

In the case of edible insects, reliable consumption data remain limited, which currently constrains robust dietary exposure assessment. Furthermore, several contaminants discussed in this review, such as Cd, acrylamide, and certain PAHs, are substances for which the concept of a completely safe exposure level may not be applicable. In these cases, risk management in food safety frameworks is generally based on minimizing exposure according to the ALARA (as low as reasonably achievable) principle (European Commission 2006; EFSA Panel on Dietetic Products, Nutrition and Allergies [NDA] 2012). Therefore, the available occurrence data should not be interpreted as direct evidence of regulatory compliance or consumer risk but rather as an initial indication of potential exposure that requires further investigation through comprehensive dietary exposure assessments.

In addition to the contaminant‐specific considerations discussed above, the literature reviewed in this study allows broader conclusions regarding the occurrence, determinants, and toxicological relevance of chemical contaminants in insects intended for human consumption.

Overall, Cd and Pb emerged as the contaminants of greatest concern due to the higher frequency of regulatory exceedances, particularly in products originating from environmentally contaminated regions or produced under less controlled conditions (Muhammad et al. 2022; Mwelwa et al. 2023; Gori et al. 2025). In contrast, Ni concentrations were generally low and rarely associated with estimated dietary exposures of toxicological concern (Truzzi et al. 2019; Sikora et al. 2023).

For process‐related contaminants, the reviewed evidence suggests that acrylamide formation is more strongly influenced by processing conditions than by the incorporation of insect‐derived ingredients themselves (Burešová et al. 2023; Bartkiene et al. 2023).

Some insect species also appeared consistently to have lower levels of heavy metal accumulation than others. Farmed species such as *T. molitor*, *A. domesticus*, *A. diaperinus*, and *B. mori* generally presented contaminant concentrations below currently applicable regulatory benchmarks, particularly when reared under controlled farming systems and standardized feeding conditions (Poma et al. 2017; Kosečková et al. 2022; Sikora et al. 2023).

Conversely, wild‐harvested insects and species collected in mining‐impacted or environmentally contaminated regions exhibited substantially higher variability and, in some cases, concentrations associated with potential toxicological concern (Badanaro et al. 2022; Mwelwa et al. 2023). Species such as scorpions and certain aquatic insects also demonstrated comparatively higher accumulation potential for Cd and Pb (Kitahara et al. 2022; Holowaty et al. 2024).

Collectively, the available evidence indicates that controlled production systems play a critical role in reducing contaminant occurrence in edible insects. Studies conducted under regulated farming conditions consistently demonstrated lower concentrations of Cd, Pb, Hg, and Ni compared with insects collected from environmentally exposed ecosystems (Hyun et al. 2012; Truzzi et al. 2019; Van Der Fels‐Klerx et al. 2020).

Feed substrate composition, environmental quality, and production chain management emerged as central determinants of contaminant transfer. These findings support the interpretation that contaminant occurrence in edible insects is predominantly driven by external environmental and production‐related factors rather than by intrinsic insect physiology alone.

Processing methods also appear to significantly influence contaminant occurrence, although the direction and magnitude of this effect vary according to the contaminant and processing technique applied.

Thermal processing, degutting, roasting, drying, and fermentation were associated with substantial reductions in Cd and Pb concentrations in some studies (Murefu et al. 2023; Bartkiene et al. 2023). Similarly, fermentation strategies demonstrated the potential to mitigate acrylamide formation in insect‐enriched bakery products (Bartkiene et al. 2023).

However, other forms of processing, particularly concentration steps leading to powders or defatted meals, may increase detectable contaminant concentrations due to water loss and matrix concentration effects, as observed in *B. mori*‐derived products and insect powders commercialized in Europe (Marzoli et al. 2023; Gori et al. 2025).

Despite the growing number of studies addressing contaminants in edible insects, the current body of evidence still presents considerable scientific uncertainty. The main limitations include the heterogeneity of analytical methodologies, differences in exposure assumptions, inconsistent use of toxicological reference values, and the absence of harmonized insect‐specific regulatory limits for most contaminants.

Furthermore, reliable consumption data for edible insects remain scarce in most populations, limiting robust dietary exposure assessment. Consequently, although the available evidence generally suggests low toxicological concern for insects produced under controlled conditions, the literature still lacks sufficient standardization to support definitive risk characterization across all species, contaminants, and production systems.

## 5 Conclusions

This review demonstrates that the occurrence and concentration levels of chemical contaminants of food safety concern, namely, metal contaminants (Cd, Pb, Hg, and Ni) and process contaminants (PAHs and acrylamide), vary considerably among edible insects and insect‐derived food products according to insect species, rearing substrate, processing conditions, and environmental factors. For Cd, Ni, Pb, and Hg, most studies report low levels in insects reared under controlled conditions, with no significant bioaccumulation observed. Studies on PAHs and acrylamide indicate that under low exposure conditions, accumulation in insects is limited, and contaminants are rapidly eliminated. However, at higher exposure levels, PAH accumulation increases and, in some cases, may exceed regulatory benchmark values. Overall, the data suggest that insects raised on appropriate substrates under controlled conditions for entomophagy generally exhibited contaminant concentrations below the comparative regulatory benchmark values for metal contaminants, PAHs, and acrylamide. These findings highlight the importance of substrate selection and monitoring contaminant levels to support compliance with applicable regulatory standards and minimize potential dietary exposure. This review provides a comprehensive overview of the occurrence of chemical contaminants in edible insects and insect‐derived food products, together with a discussion of their implications for food safety and future risk assessment. The available evidence indicates that contaminant occurrence in edible insects should be interpreted within an exposure‐based risk assessment framework that distinguishes hazard identification from toxicological risk and regulatory compliance. Continued generation of harmonized occurrence data, improved consumption estimates for insect‐based foods, and the establishment of insect‐specific regulatory benchmarks will strengthen future exposure‐based contaminant risk assessments and support evidence‐based regulatory development for insect‐based foods.

## Author Contributions

**Marta Esgalhado**: conceptualization, investigation, writing – review and editing. **Dayanne da Costa Maynard**: conceptualization, methodology, software, investigation, writing – review and editing. **Ariana Saraiva**: conceptualization, investigation, writing – review and editing. **Marjhory Gonçalves Moura**: investigation, writing – review and editing. **Didem Aybike Haspolat**: investigation, writing – review and editing. **José Costa**: investigation, writing – review and editing. **José Manuel Lorenzo**: validation. **Zsuzsa Farkas**: investigation, validation, resources, project administration, writing – review and editing. **António Raposo**: conceptualization, investigation, validation, writing – review and editing, supervision, funding acquisition, project administration. **Maria do Céu Costa**: conceptualization, investigation, validation, writing – original draft, resources, writing – review and editing, supervision, funding acquisition, project administration.

## Funding

CONTEMFood: Contaminants in Meat Replacement Products: A Contemporary Food Safety Perspective 2024‐1.2.3‐HU‐RIZONT‐2024‐00098 (2025/01/01–2027/12/31); Fundação para a Ciência e a Tecnologia, I.P. (FCT), under Grant UID/04567/2025 (https://doi.org/10.54499/UID/04567/2025).

## Conflicts of Interest

The authors declare no conflicts of interest.

## 1

**TABLE A1:** Search strategies.

PubMed
(“edible insect” [Title/Abstract] OR “edible insects” [Title/Abstract] OR “insect protein” [Title/Abstract] OR “insects protein” [Title/Abstract] OR “entomophagy” [Title/Abstract] OR “insect consumption” [Title/Abstract] OR “insects consumption” [Title/Abstract]) AND ((“mercury” OR “cadmium” OR “nickel” OR “lead”) OR (“PAH” OR “polycyclic aromatic hydrocarbons”) OR (“acrylamide”)
Scopus
TITLE‐ABS‐KEY ((“edible insect” OR “edible insects” OR “insect protein” OR “insects protein” OR “entomophagy” OR “insect consumption” OR “insects consumption”) AND (mercury OR Hg OR cadmium OR Cd OR nickel OR Ni OR lead OR Pb OR PAH OR PAHs OR “polycyclic aromatic hydrocarbon” OR “polycyclic aromatic hydrocarbons” OR acrylamide))
Google Scholar
(intitle: “edible insect” OR intitle: “edible insects” OR intitle: “entomophagy” OR intitle: “insect protein” OR intitle: “insects protein” OR intitle: “insect consumption” OR intitle: “insects consumption”) AND (mercury OR Hg OR cadmium OR Cd OR nickel OR Ni OR lead OR Pb OR PAH OR PAHs OR “polycyclic aromatic hydrocarbon” OR “polycyclic aromatic hydrocarbons” OR acrylamide)

**TABLE A2:** Data extraction table.

	Year	Author	Country of the study	Title	Objective	Sample type	Species	Origin	Target contaminant(s)	Analytical method	Main results	Referenced legal
1	2009	Zhang et al.	China	Mercury, cadmium and lead biogeochemistry in the soil–plant–insect system in Huludao City	To investigate and compare the concentrations of mercury (Hg), cadmium (Cd), and lead (Pb) in ashed plant and insect samples with those in soil, to understand their biogeochemical behavior and transfer along food chains in Huludao City	Whole insect	*Locusta migratoria manilensis*, *Acrida chinensis*, *Holotrichia* spp., *Eligma narcissus* larvae, mantis, spider and dragonfly	In Wuli River and Cishan River	Cd, Pb, Hg	CVAAS, CVAFS, ICPS, ICP–MS	Among the four herbivorous insects studied, *E. narcissus* larvae showed the highest metal concentrations, while *L. migratoria manilensis* had the lowest. *Holotrichia* exhibited slightly lower average mercury, cadmium, and lead levels than *E. narcissus* larvae	No legal regulations were referenced in the study
2	2012	Hyun et al.	South Korea	Evaluation of nutritional status of an edible grasshopper, *Oxya chinensis formosana*	To examine the nutritional status of the grasshopper (*O. chinensis formosana*) as a potential human food source and to evaluate its suitability as an alternative edible resource	Insect powder	*O. chinensis formosana* (grasshopper)	In South Kora	Cd, Pb, Hg	AAS	Low concentrations of Pb, Cd, and Hg were detected in *O. chinensis formosana*, with all reported concentrations remaining below the comparative regulatory benchmark values referenced in the study	No legal regulations were referenced in the study
3	2015	Charlton et al.	United Kingdom	Exploring the chemical contaminant profile of fly larvae as a source of protein for animal feed	To assess the chemical contaminant profile of larvae from four fly species reared under different conditions and locations by measuring key contaminants (Cd, Pb, Hg, and Ni, pesticides, dioxins, and mycotoxins) and evaluating their contaminant levels against applicable regulatory benchmark values for animal feed	Whole insect	Fly larvae (*Musca domestica*, *Calliphora vomitoria*, *Chrysomya* spp. and *Hermetia illucens*)	In UK, China, and Ghana	Cd, PAH	ICP–MS	Cd concentrations in *M. domestica* larvae exceeded EU feed limits, whereas other species complied. PAH4 levels showed variability, with some samples exceeding the comparative regulatory benchmark values, influenced by species, substrate, and origin	Directive 2002/32/EC Commission Regulation (EU) No 835/2011
4	2017	Poma et al.	Belgium	Evaluation of hazardous chemicals in edible insects and insect‐based food intended for human consumption	To investigate organic contaminants (flame retardants, PCBs, DDT, dioxins, pesticides) and metals in selected edible insects and insect‐based food products in Belgium	Whole insect and insect‐based products	*Galleria mellonella*, *L. migratoria*, *Tenebrio molitor*, *Alphitobius diaperinus*	In Belgium	Cd, Ni, Pb	ICP–MS	Low levels of Pb, Ni, and Cd were detected in insect species and insect‐based products in Belgium, with concentrations comparable to those reported for conventional animal‐derived foods	Commission Regulation (EU) 2023/915
5	2019	Köhler et al.	Thailand	Protein, amino acid and mineral composition of some edible insects from Thailand	To assess protein content, amino acid composition, and mineral profile of selected edible insects from Thailand, expressed on a fresh weight basis its suitability for human nutrition assessment, food labeling, and inclusion in food composition databases, thereby supporting the promotion and acceptance of edible insects as a food resource	Insect‐based products	*Patanga succincta* (Bombay locust), *Holotrichia* sp. (scarab beetle), *Acheta domesticus* (house cricket), and *Bombyx mori* (mulberry silkworm)	From a supermarket in downtown Bangkok	Cd, Pb, Hg	ICP–MS, CV‐AAS	Very low Cd and Hg levels were detected across all insect samples, while Pb ranged from 0.0044 to 0.0155 mg/100 g, with the highest concentration observed in street‐vendor house crickets	Directive 2002/31/EC
6	2019	Truzzi et al.	Italy	Influence of feeding substrates on the presence of toxic metals (Cd, Pb, Ni, As, Hg) in larvae of *Tenebrio molitor*: Risk assessment for human consumption	To assess metal levels in *T. molitor* larvae, examine the effect of rearing substrates on accumulation, and evaluate associated human health risks	Whole insect	*T. molitor*	From a pet shop in Italy	Cd, Ni, Pb, Hg	GF‐AAS, TDA‐AAS	Low accumulation of Cd and Ni was observed, Pb levels were below the comparative EU regulatory benchmark values, and Hg showed bioaccumulation; overall results indicated limited dietary exposure under the reported conditions	Commission Regulation (EU) 2023/915 Commission Regulation (EU) No 420/2011 Council Directive 2002/32/EC
7	2020	Kim et al.	South Korea	Comparative analysis of nutrients and hazardous substances in *L. migratoria* from host plants	To evaluate the nutritional composition and contaminant and microbiological profile of *L. migratoria* as an edible insect by analyzing and comparing its nutrient content, Cd, Pb, Hg, and Ni, and pathogens when reared on different host plants (corn and wheat)	Insect powder	*L. migratoria*	From farms, reared in South Korea	Cd, Pb, Hg	ICP‒OES	Feeding substrate influenced metal concentrations in *L. migratoria*, particularly for Cd and Pb, while Hg remained consistently low; all measured levels remained below the comparative regulatory benchmark values for edible insects referenced in the study	Korean regulatory limits for edible insects/feed contaminants (general national guideline values)
8	2020	Devkota and Schmidt	Greece	Accumulation of heavy metals in food plants and grasshoppers from the Taigetos Mountains, Greece	To assess heavy metal accumulation in plants and grasshoppers and their transfer in the terrestrial food chain of the Taigetos Mountains	Whole insect	*Calliptamus italicus*, *Oedipoda caerulescens*, *Oedipoda germanica*, *Chorthippus (Glyptobothrus) crassiceps*	From mountainous grassland ecosystems in the Taigetos Mountains	Cd, Pb, Hg	AAS	Grasshoppers showed lower Pb levels than host plants, indicating no biomagnification, whereas elevated Hg concentrations suggested potential bioaccumulation and trophic transfer	No legal regulations were referenced in the study
9	2020	Fels‐Klerx et al.	the Netherlands	Chemical contaminants in black soldier fly larvae (*H. illucens*) reared on former foodstuffs for feed and food use	To assess chemical contaminant accumulation in *H. illucens* larvae reared on former food products and compare contaminant concentrations with applicable regulatory benchmark values for feed and food applications	Insect powder	Black soldier fly (*H. illucens*)	In the Netherlands	Cd, Pb, Hg, PAH	ETAAS, CV‐AFS, GC‐HRMS	Low levels of Pb, Hg, and PAHs were observed, with limited accumulation in larvae and concentrations remaining below the comparative EU regulatory benchmark values, indicating limited contaminant accumulation in the insect production system	Council Directive 2002/32/EC
10	2021	Soren et al.	India	Nutrient and toxic heavy metal assessment of *Tarbinskiellus portentosus* and *Schizodactylus monstrosus* consumed by the Bodo tribe in Assam, India	To assess the nutritional profile and heavy metal levels of *T. portentosus* and *S. monstrosus* and compare heavy metal concentrations with applicable regulatory benchmark values	Insect powder	*T. portentosus* and *S. monstrosus*	From local markets in Kokrajhar	Cd, Pb	ICP‒OES	Cd and Pb levels differed between species, with higher Pb in *T. portentosus* and generally low Cd in both species, indicating species‐dependent accumulation, with concentrations compared against applicable regulatory benchmark values	No legal regulations were referenced in the study
11	2021	Kolakowski et al.	Canada	Analysis of microbiological and chemical hazards in edible insects available to Canadian consumers	To assess microbiological contamination and chemical residues in retail and online insect‐based foods in Canada	Whole insect, insect‐based products and insect powder	Crickets, silkworm	From the Canadian retail market, online retailers and physical retail stores	Cd, Pb, Hg	ICP–MS, CV‐AFS	Measurable Cd, Pb, and Hg levels were detected in cricket‐based products, with higher accumulation in crickets than silkworm pupae; however, all concentrations remained below the comparative Canadian regulatory benchmark values, indicating limited dietary exposure under the reported conditions	Canada, Health (2005a)
12	2022	Kosečková et al.	Czech Republic	Mineral profile of cricket powders, some edible insect species and their implication for gastronomy	To assess mineral composition of cricket powder and selected edible insects, their contribution to dietary reference values, and their potential for food enrichment	Insect powder	*A. domesticus* (house cricket), yellow mealworm beetle larvae, desert locust, superworm larva	From retail supermarkets and pet shops in the south Moravian region, Czech Republic	Cd, Ni, Pb	HR‐CS, GF‐AAS	Low concentrations of Cd, Ni, and Pb were observed in edible insects, all below EU regulatory limits, with concentrations remaining below the comparative EU regulatory benchmark values	Commission Regulation (EU) 2023/915
13	2022	Kitahara et al.	Japan	Survey of arsenic/heavy metals and pesticide residues in edible insects for human consumption or supplied in Japan	To assess arsenic, heavy metal, and pesticide contamination in edible insects in Japan in relation to applicable regulatory benchmark values	Insect powder	Beetles, crickets, silkworms, Sago worms, scorpions	From locally available edible insect products (domestic market)	Cd, Pb, Hg	ICP–MS	Cd, Pb, and Hg were commonly detected in insect samples at low concentrations, and despite their occurrence, overall dietary exposure was limited under the reported consumption conditions	Regulation (EU) 2015/2283 on novel foods
14	2022	Muhammad et al.	Nigeria	Determination of heavy metals (Co, Cu, Cd, Fe, Pb, Zn) in some edible insects and fingerlings in Dutsın‐Ma Town	To evaluate heavy metal levels in selected insects and assess associated human health risks using FAO/WHO standards	Insect powder	*Zonocerus variegatus* (grasshopper), *Schistocerca gregaria* (locust), *Macrotermes bellicosus* (termite)	From Dutsin‐Ma, Katsina State, Nigeria	Cd, Pb	AAS	Species‐dependent variation was observed in Cd concentrations, with elevated levels in *Z. variegatus*, whereas Pb levels remained below permissible limits; Cd concentrations were discussed by the authors in relation to potential chronic dietary exposure	FAO/WHO
15	2022	Badanaro et al.	Togo	Sustainable local food evaluation by dosage of some metallic pollutants in wild insect species consumed in Togo	The objective of this work was to evaluate the accumulation levels of trace metal elements (TMEs) in 12 commonly consumed adult insect species collected across Togo, and to assess the associated human health risks	Insect powder	Coleoptera (*Cybister tripunctatus*, *Gnathocera trivittata*, *Gnathocera impressa*, *Gnathocera varians*, *Rhabdotis sobrina*, *Pachnoda cordata*, *Pachnoda marginata*, *Sternocera interrupta*), Isoptera (*M. bellicosus*) and Orthoptera (*Acanthacris ruficornis*, *Kraussaria angulifera*, *Brachytrupes membranaceus)*	From different ecosystems including croplands, forests, savannas, and aquatic environments	Cd, Pb, Hg	ICP–MS	Most species complied with EU limits for Cd, Pb, and Hg, although *C. tripunctatus* showed slight exceedance of Cd and Pb, with generally higher accumulation in aquatic compared to terrestrial species	Commission Regulation (EU) 2023/915
16	2023	Sikora et al.	Poland	Elemental content of the commercial insect‐based products available in the European Union	To assess the elemental composition of insect‐based food and feed products in the European Union in relation to nutritional value and consumer risk	Whole insect, insect‐based products, insect powder	Buffalo worm *A. diaperinus*, yellow mealworm *T. molitor*, house cricket *A. domesticus*, migratory locust *L. migratoria*, domesticated silkworm *B. mori*	From online stores registered in the European Union	Cd, Ni, Pb, Hg	ICP–OES	Heavy metal concentrations (Cd, Ni, Pb, and Hg) in insect‐based food and feed products were generally low and compliant with regulatory limits, except for a single Pb outlier, indicating limited contaminant occurrence	Commission Regulation (EU) 2021/1317
17	2023	Mwelwa et al.	Zambia	Data to understand the biotransfer of heavy metals along the soil‒plant‐edible insect‐human food chain in Africa/biotransfer of heavy metals along the soil‒plant‐edible insect‐human food chain in Africa	To evaluate heavy metal transfer along a soil–plant–edible insect food chain and assess human health risks using daily intake and THQ	Whole insect and insect powder	*Cyrtacanthacris forda*, *Imbrasia obscura*, *Imbrasia rubra*, *Imbrasia epimethea*, *Macrotermes falciger* (termite), *Ruspolia differens*	In Africa, specifically in the Copperbelt Province of Zambia	Cd, Ni, Pb	AAS	Cd and Ni concentrations decreased along the pollution gradient in soils and insects, whereas Pb showed higher levels in host plants and species‐dependent variation, indicating strong environmental and biological control of accumulation	City region food system situational analysis. Kitwe, Zambia FAO
18	2023	Marzoli et al.	Italy	Microbiological and chemical contaminant profile of *Bombyx mori* farmed in north‐eastern Italy as a novel food source	To assess the nutritional composition and chemical contaminant and microbiological profiles of *B. mori* and its derived products in Italy for food use evaluation	Whole insect and insect powder	*B. mori*	From rearing farms in Veneto Region, Italy	Cd, Pb, Hg	ICP–MS	Cd and Pb were detected across all samples, with the highest concentrations in defatted chrysalis meal, although contaminant concentrations remained below the comparative regulatory benchmark values	Commission Implementing Regulation (EU) 2017/2470
19	2023	Burešová et al.	Lithuania	Does the addition of edible insects affect the formation of acrylamide during bread baking?	The aim of this study was to investigate the effect of insect powder addition on acrylamide formation in leavened and unleavened wheat bread and on the content of its main precursors in raw materials	Insect powder	*A. domesticus* and *T. molitor*	From under controlled laboratory baking conditions	Acrylamide	LC‒MS/MS, HPLC	Insect flours increased reducing sugars and amino acids while lowering asparagine, and showed mixed effects on acrylamide formation depending on bread type; overall, they improved nutritional quality while maintaining acrylamide concentrations below the comparative regulatory benchmark values	Regulation (EU) 2015/2283
20	2023	Bartkiene et al.	Lithuania	Crickets (*Acheta domesticus*) as wheat bread ingredient: Influence on bread quality and acrylamide formation	To assess consumer attitudes toward edible insects and evaluate the impact of cricket flour on wheat bread quality and acrylamide formation. The study applied fermentation with *Lactiplantibacillus plantarum* No. 122 to reduce acrylamide formation in bread	Insect powder	*A. domesticus*	In Lithuania	Acrylamide	GC–ECD	Fermentation significantly reduced acrylamide formation in breads with 10% and 20% cricket flour, with no significant effect at 30%, while maintaining product quality	Regulation (EU) 2015/2283
21	2023	Murefu et al.	Zimbabwe	Effects of post‐harvesting practices on heavy metal levels of mopane caterpillar (*Gonimbrasia belina*) products and associated risk assessment	To assess how post‐harvest processing methods influence heavy metal levels in mopane caterpillars and evaluate associated consumer health risks	Whole insect	Mopane caterpillar (*G. belina*)	From households in Gwanda District, Zimbabwe	Cd, Ni, Pb, Hg	AAS	In *G. belina*, heavy metal concentrations were low and significantly affected by processing, with Cd and Pb reduced after treatment and Hg and Ni not detected, indicating that processing effectively reduced heavy metal concentrations and potential dietary exposure	EFSA Scientific Committee (2015)
22	2024	Machado et al.	Czech Republic	Bioaccessibility of trace elements and Fe and Al endogenic nanoparticles in farmed insects: Pursuing quality sustainable food	To assess the in vitro bioaccessibility of selected elements in three farmed edible insect species	Whole insect	*A. domesticus* (house cricket), *L. migratoria* (migratory locust), *T. molitor* (yellow mealworm larva)	From the rearing facility of the Czech Republic	Pb	CP–MS	Pb levels differed among insect species, with all values remaining below the comparative EU regulatory benchmark values and significant interspecific variation, particularly between *T. molitor* and *A. domesticus*, likely due to biological and environmental factors	Commission Regulation (EU) 2023/915
23	2024	Jankauskienė et al.	Lithuania	The impact of freeze‐dried *Tenebrio molitor* larvae on the quality, acrylamide formation, and sensory acceptability of wheat bread	To evaluate the impact of *T. molitor* powder incorporation on wheat bread quality, nutritional value, and consumer acceptance	Insect powder	*T. molitor*	In Vilnius, Lithuania	Acrylamide	GC–ECD	Acrylamide levels in all breads, including those with mealworm powder, remained below the regulatory benchmark values used as comparative references, with no consistent effect of inclusion level and overall nutritional improvement	Commission Regulation (EU) 2017/2158
24	2024	Holowaty et al.	France	Multi‐elemental analysis of edible insects, scorpions, and tarantulas from french (online) market and human health risk assessment due to their consumption: A pilot study	To develop and validate an ICP–MS method for multielement analysis of edible insects using microwave digestion	Whole insect	*A. domesticus*, *T. molitor*, *Atta laevigata*, Nepidae, *L. migratoria*, *Heterometrus longimanus*, and *Haplopelma albostriatum*	In French retail market and online	Cd, Pb	ICP–MS/MS	Pb and Cd levels showed clear species‐dependent variation. Pb concentrations were lowest in mealworms (20.9 µg/kg) and crickets (41.6 µg/kg), followed by locusts (61.8 µg/kg), water bugs (82.93 µg/kg), ants (105.5 µg/kg), and scorpions (146.3 µg/kg), with the highest reported value reaching 556.6 µg/kg in an unspecified species.	FAO/WHO
											Cd levels showed a similar pattern, with the lowest accumulation observed in mealworms, crickets, and locusts. Overall, both Pb and Cd were lowest in mealworms, crickets, and locusts, while higher levels were found in scorpions and certain other insect groups. This indicates that heavy metal accumulation can vary significantly depending on the insect species	
25	2025	Gori et al.	Italy	Heavy metals (Pb, Cd, Ni) in insect‐based products for human consumption sold by E‐commerce in the EU market: Occurrence and potential health risk associated with dietary exposure	To determine Pb, Cd, and Ni levels in insect‐based products sold online in Europe and assess potential human health risks using EFSA reference values	Insects‐based products	*A. diaperinus*, *A. domesticus*, *T. molitor*, *L. migratoria*	Commercialized in the EU	Cd, Ni, Pb	GFAAS	Pb, Cd, and Ni levels in insect‐based products varied by composition and species, with some Pb and Cd exceedance but overall exposure below EFSA thresholds; findings highlight the need for monitoring heavy metal levels	Commission Regulation (EU) 2023/915 Commission Regulation (EU) 2023/58 Commission Regulation (EU) 2022/188 Commission Regulation (EU) 2023/5 Commission Regulation (EU) 2021/1975 Commission Regulation (EU) 2021/882 Commission Regulation (EU) 2022/169
